# Analyzing dynamic species abundance distributions using generalized linear mixed models

**DOI:** 10.1002/ecy.3742

**Published:** 2022-06-23

**Authors:** Erik Blystad Solbu, Bert van der Veen, Ivar Herfindal, Knut Anders Hovstad

**Affiliations:** ^1^ Department of Landscape and Biodiversity Norwegian Institute of Bioeconomy Research (NIBIO) Trondheim Norway; ^2^ Department of Mathematics Norwegian University of Science and Technology (NTNU) Trondheim Norway; ^3^ Centre for Biodiversity Dynamics, Department of Biology Norwegian University of Science and Technology (NTNU) Trondheim Norway; ^4^ Norwegian Biodiversity Information Centre Trondheim Norway

**Keywords:** environmental variance, generalized linear mixed model, Poisson lognormal, population dynamics, spatial and temporal correlation, species abundance distribution, species heterogeneity, variance partitioning

## Abstract

Understanding the mechanisms of ecological community dynamics and how they could be affected by environmental changes is important. Population dynamic models have well known ecological parameters that describe key characteristics of species such as the effect of environmental noise and demographic variance on the dynamics, the long‐term growth rate, and strength of density regulation. These parameters are also central for detecting and understanding changes in communities of species; however, incorporating such vital parameters into models of community dynamics is challenging. In this paper, we demonstrate how generalized linear mixed models specified as intercept‐only models with different random effects can be used to fit dynamic species abundance distributions. Each random effect has an ecologically meaningful interpretation either describing general and species‐specific responses to environmental stochasticity in time or space, or variation in growth rate and carrying capacity among species. We use simulations to show that the accuracy of the estimation depends on the strength of density regulation in discrete population dynamics. The estimation of different covariance and population dynamic parameters, with corresponding statistical uncertainties, is demonstrated for case studies of fish and bat communities. We find that species heterogeneity is the main factor of spatial and temporal community similarity for both case studies.

## INTRODUCTION

Human activities are causing biodiversity declines at unprecedented rates (Brondizio et al., 2019). The human factors affecting biodiversity patterns, which vary in time and space, combined with complex dynamics of species communities, make it challenging to acquire knowledge on the ecological mechanisms involved in biodiversity change. However, it is vital to obtain a better understanding of the natural and anthropogenic processes causing biodiversity change to initiate actions to reduce and reverse biodiversity loss (Davison et al., 2021; Naeem et al., 2012; Sutherland et al., 2004). Ecological models that quantitatively relate biodiversity changes to ecological processes will improve our understanding of changes in species communities.

One approach to studying community dynamics by Engen and Lande (1996b) showed how population dynamic models can generate species abundance distributions. Species abundance distributions describe the differences in abundance of species in a community (McGill, 2011), which could now be explained by population dynamical processes. Specifically, Engen and Lande (1996a, 1996b) and later Diserud and Engen (2000) showed how the species' growth rates, shapes and strengths of density regulation, carrying capacities, responses to environmental and demographic stochasticity, all determine the mean and variance of the species abundance distribution. The spatio‐temporal analysis of communities using the dynamic species abundance distribution was explained in Engen et al. (2002).

The variance of the dynamic species abundance distribution can be partitioned into several different well known components of population dynamics (Engen et al., 2002). The variance partitioning consists of environmental noise, demographic stochasticity, species heterogeneity, expressed as variance in log carrying capacity between species, and sampling variation. For instance, Bellier et al. (2014) found that the spatio‐temporal environmental variance accounted for 75% of the variation in a freshwater zooplankton community, whereas in a tropical butterfly community the species heterogeneity accounted for 81% of the variance (Engen et al., 2002). This is critical information for conservation, as large variation in species heterogeneity indicates that many species have low levels of carrying capacity, which increases their extinction risk (Henle et al., 2004).

The purpose of this paper was to describe how standard general linear mixed models (GLMMs) (Bolker et al., 2009; Harrison et al., 2018) can be used to model dynamic species abundance distributions, and to partition the variance of the abundance distribution into several components with a well defined ecological meaning. By doing so, we can relate population dynamic theory to a modeling framework that is available, accessible, and familiar to many ecologists. First we revisit the theory of dynamic species abundance distributions, and show how the population dynamic model generates different components of variation in relative and mean log abundance of species. We then define a GLMM with variance components that correspond to the variance of the dynamic species abundance distribution. With the connection between dynamic species abundance distribution and GLMM established, we investigate how different community dynamics can affect our estimation with a simulation study. Finally, we illustrate how community dynamics and spatial and temporal correlation is analyzed for two community data sets found in an open‐access database.

## METHODS

### Dynamic species abundance distributions

Let *X*_*i*_ be the log abundance of species *i*, we assume the dynamics of *X*_*i*_ can be described by a stochastic differential equation

(1) $$dX_{i}=\left[r_{i}-\gamma X_{i}\right]dt+\sigma_{s}dB_{i}\left(t\right)+\sigma_{E}dE\left(t\right),$$

where we have ignored among‐species density dependence and demographic variance for brevity (please refer to equation 8.1b in Lande et al. (2003) for more details regarding the extended process). The density independent long‐run growth rate *r*_*i*_ of species *i* is assumed to be normally distributed among species with mean *μ*_*r*_ and variance $\sigma_{r}^{2}$. The species‐specific strength of density dependence γ is assumed to be the same for all species and has a Gompertz (log–linear) form. The general response to environmental variability, i.e., the response shared for all species, is described by $\sigma_{E}^{2}$ and the species‐specific response by $\sigma_{s}^{2}$. Finally, $dB_{i}\left(t\right)$ and d*E*(*t*) are Brownian motions that are independent, identically distributed, and follow a normal distribution with mean zero and variance d*t*. All notation introduced above and in the following paragraphs is summarized in Appendix S1.

The continuous time process in Equation (1) is known as a diffusion process, and has two properties describing the population dynamics. The first property can be thought of as expected change in abundance, describing deterministic dynamics, and is denoted by $r_{i}-\gamma X_{i}$ for species *i*. The second property describes random fluctuations, denoted by $\sigma_{s}^{2}$ and $\sigma_{E}^{2}$ for species *i*. Both properties are described over a very short time interval and are known as infinitesimal mean and infinitesimal variance, respectively (Czuppon & Traulsen, 2021). If the infinitesimal mean is linear, e.g., $M\left(dX\right)=a+\mathrm{bX}$, and the variance is constant, *V*(d*X*) = *c*, the process has a stationary distribution that describes the probability distribution of $X_{i}\left(t\right)$ when the effect of its initial value has diminished. This stationary distribution is normal with mean $\mu_{X}=a/b$ and variance $\sigma_{X}^{2}=c/2b$. It can be shown that the mean log abundance $\bar{X}=\frac{1}{S}\sum_{i=1}^{s}X_{i}$ and the relative log abundance $x_{i}=X_{i}-\bar{X}$ are also diffusion processes (Lande et al., 2003). Assuming that the number of species *S* is large so that $\frac{1}{S}\approx 0$, the diffusion processes of *x*_*i*_ and $\bar{X}$ have linear infinitesimal means, and constant infinitesimal variances.

The stationary distribution of relative abundance *x*_*i*_ has mean $\left(r_{i}-\mu_{r}\right)/\gamma$ and variance $\sigma_{s}^{2}/2\gamma$, and the mean log abundance $\bar{X}$ has mean $\bar{r}/\gamma$ and variance $\sigma_{E}^{2}/2\gamma$, where $\bar{r}=\frac{1}{S}\sum_{i=1}^{s}r_{i}$. Because *r*_*i*_ is also normally distributed, the species abundance distribution of relative log abundances is normal with mean zero and variance

(2) $$\mathrm{var}\left[x_{i}\left(t\right)\right]=\frac{\sigma_{s}^{2}}{2\gamma }+\frac{\sigma_{r}^{2}}{\gamma^{2}}.$$

From the description above, the distribution of log abundances *X*_*i*_, can be obtained by reformulating it as $X_{i}=X_{i}-\bar{X}+\bar{X}=x_{i}+\bar{X}$, which from the diffusion processes has a normal distribution with mean $\bar{r}/\gamma$ and variance $\mathrm{var}\left[X_{i}\left(t\right)\right]=\sigma_{r}^{2}/\gamma^{2}+\sigma_{s}^{2}/2\gamma +\sigma_{E}^{2}/2\gamma$. The temporal covariation of time interval *u*, follows from properties of the diffusion processes and is

(3) $$\mathrm{cov}\left[X_{i}\left(t\right),X_{i}\left(t+u\right)\right]=\frac{\sigma_{r}^{2}}{\gamma^{2}}+\frac{\sigma_{s}^{2}}{2\gamma }e^{-\gamma u}+\frac{\sigma_{E}^{2}}{2\gamma }e^{-\gamma u}.$$

In the next section we show how the same covariance function can be defined in a GLMM framework.

### GLMM formulation

In a model that describes spatial and temporal dynamics in an ecological community, the response $y_{\mathrm{ij}}\left(t,z\right)$ is typically the abundance of a species *i*, at time *t* and location *z*, and observation replicate *j* (if applicable). The abundance is measured as counts, and the observational distribution is assumed to be $\text{Poisson}\left[\mu_{\mathrm{ij}}\left(t,z\right)\right]$, with log‐link $\eta_{\mathrm{ij}}\left(t,z\right)=\log\left[\mu_{\mathrm{ij}}\left(t,z\right)\right]$. We simplify our analysis by assuming that temporal (*t*) and spatial (*z*) dynamics can be modeled separately. This separation of dimensions means that we fit *two* models if both dimensions are represented in the data set. In the following we consider temporal dynamics, but the same results can be used for spatial dynamics.

For a community studied over time, the linear predictor $\eta =\left[\eta_{\mathrm{ij}}\left(t\right)\right]$ is

(4) $$\eta =X\;\beta +\mathrm{Zb}=1\beta_{0}+\left[Z_{h}\;Z_{e}\;Z_{c}\;Z_{o}\right]\left[\begin{array}{c} b_{h}\\ b_{e}\\ b_{c}\\ b_{o} \end{array}\right],$$

where ***X*** is the design‐matrix for fixed effects, and **β** is the vector of fixed effects. In all cases considered here, we assume $X=1_{N}$, and $\beta =\beta_{0}$, where $1_{N}$ is an N×1 vector of 1 s and β_0_ is a scalar. The total number of observations *N* is the product of the number of species *S*, time points *T* and repeated observations *J*.

The design‐matrix **Z** for random effects is split into four different components (their ecological interpretation is explained in the next section). These are most compactly described using Kronecker products, ‘⊗’, meaning that we multiply each element of the matrix to the left of the product with the whole matrix to the right. The first matrix, $Z_{h}=I_{S}\;⊗\;1_{T}\;⊗\;1_{J}$, where $I_{S}$ is a diagonal matrix of order *S*, assigns one random effect to each species. The second matrix, $Z_{e}=I_{S}\;⊗\;I_{T}\;⊗\;1_{J}$, assigns one random effect to each species at each time point. The third matrix, $Z_{c}=1_{S}\;⊗\;I_{T}\;⊗\;1_{J}$ assigns one random effect to each time point, and finally $Z_{o}=I_{S}\;⊗\;I_{T}\;⊗\;I_{J}$ assigns one random effect to each observation. Similarly, the vector of random effects ***b*** has four components: $b_{h}=\left[h_{i}\right]$ is a S×1 vector of among‐species effects, $b_{e}=\left[e_{i}\left(t\right)\right]$ is a ST×1 vector of within‐species effects, $b_{c}=\left[c\left(t\right)\right]$ is a T×1 vector of among‐time effects, and $b_{o}=\left[\varepsilon_{\mathrm{ij}}\left(t\right)\right]$ is a STJ×1 vector of observation‐level effects.

The distribution of the random effects is

(5) $$b=\left[\begin{array}{c} b_{h}\\ b_{e}\\ b_{c}\\ b_{o} \end{array}\right]∼N\left(\left[\begin{array}{c} 0\\ 0\\ 0\\ 0 \end{array}\right],\left[\begin{array}{cccc} \sigma_{h}^{2}I_{S} & & & \\ & \sigma_{e}^{2}I_{S}\;⊗\;\rho & & \\ & & \sigma_{c}^{2}\rho & \\ & & & \sigma_{o}^{2}I_{S}\;⊗\;I_{T}\;⊗\;I_{J} \end{array}\right]\right),$$

where $\sigma_{h}^{2}$ is the among‐species variation, $\sigma_{e}^{2}$ is the within‐species variation, and **ρ** is a *T* × *T* symmetric matrix with elements $\rho_{\mathrm{kl}}=e^{-\gamma |t_{l}-t_{k}|}$, for any k,l∈1,…,T and ‘∣∣’ is the absolute value. Furthermore, $\sigma_{c}^{2}$ is the among‐time variation, and $\sigma_{o}^{2}$ is the observation‐level variation. From the definitions above, we can write the elements of the linear predictor **η** as

(6) $$\eta_{\mathrm{ij}}\left(t\right)=\beta_{0}+h_{i}+c\left(t\right)+e_{i}\left(t\right)+\varepsilon_{\mathrm{ij}}\left(t\right),$$

with expectation $E\left[\eta_{\mathrm{ij}}\left(t\right)\right]=\beta_{0}$, and covariance

(7) $$\mathrm{cov}\left[\eta_{\mathrm{ij}}\left(t\right),\eta_{\mathrm{kl}}\left(t+u\right)\right]=\left\{\begin{array}{ll} \sigma_{h}^{2}+\sigma_{e}^{2}+\sigma_{c}^{2}+\sigma_{o}^{2} & \;\text{for}\;i=k,j=l\;\text{and}\;u=0\\ \sigma_{h}^{2}+\sigma_{e}^{2}e^{-\mathrm{\gamma u}}+\sigma_{c}^{2}e^{-\mathrm{\gamma u}} & \;\text{for}\;i=k\;\text{and}\;j\neq l\\ \sigma_{c}^{2}e^{-\gamma u} & \;\text{for}\;i\neq k. \end{array}\right.$$

We see that the covariance between linear predictors of the same species at different time points (second line in Equation 7) has the same form as the covariance of log abundances in Equation (3), with $\sigma_{h}^{2}=\sigma_{r}^{2}/\gamma^{2},\;\sigma_{e}^{2}=\sigma_{s}^{2}/2\gamma$, and $\sigma_{c}^{2}=\sigma_{E}^{2}/2\gamma$. The correspondence between the dynamic species abundance distribution (Equation 3) and the GLMM model (Equation 7) shows that the different random effects have direct relations to population dynamics, which we will cover in the next section.

We stated above that we analyzed spatial and temporal dynamics separately. When having only spatial data, the results above remain the same by replacing “*t*” with “*z*,” “*T*” with the number of locations “*L*” and the correlation **ρ** has elements $e^{-\alpha d\left(z_{l},z_{k}\right)}$, where α is the inverse spatial scaling, 1/α, and $d\left(z_{l},z_{k}\right)$ is the distance between *z*_*k*_ and *z*_*l*_ for any k,l∈1,…,L. If we have both time points and locations, the separation of dimensions into two models mean that we group observations by location when fitting the temporal model, and group by time points when fitting the spatial model. We return to the separate models in the case studies, where we have both space and time as dimensions.

### Interpretation of intercept and random effects

Because the observational unit is counts and we use the log‐link function, the intercept β_0_ is the expected log abundance of species in the community. In most ecological studies, only a fraction of the true abundance is sampled so that the intercept β_0_ is confounded with the sampling intensity, *ν*, and on the log scale we estimate ${\tilde{\beta }}_{0}=\ln\nu +\beta_{0}$. If the proportion of the community that has been sampled is known approximately, the estimated intercept can be corrected accordingly. Under the assumption of density regulation, the intercept is equal or proportional to the average log carrying capacity r/γ=lnK over all species, locations and times (Engen et al., 2002).

**FIGURE 1:**
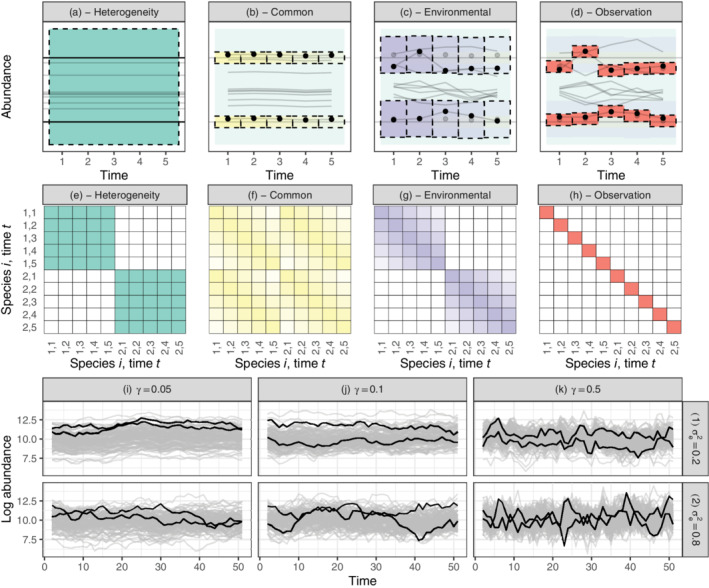
Population dynamics and covariance structure of random effects. (a–d) Schematic illustration of population dynamic interpretation of random effects. Two highlighted (black) species are shown changing throughout. Each square marked by dashed lines is a random effect, indicating a 95% probability interval around the mean. Going from (a) to (d), a new random effect is added from the previous figure. (a) Among‐species variation or species heterogeneity, $\sigma_{h}^{2}$. The horizontal lines are 10 species' carrying capacities. (b) Common environmental variance $\sigma_{c}^{2}$, synchronizing fluctuations among species. A small random effect, correlated among years (yellow shading), is added to the sampled carrying capacities from (a). (c) Within‐species environmental variation $\sigma_{e}^{2}$. For each species, a random effect, correlated among years (purple shading), is added to each time point. The gray dots are the previous values of the highlighted species. (d) Observation‐level random noise $\sigma_{o}^{2}$. This is similar to (b), but now an independent effect is added to each species within each year. (e–h) Covariance matrix for linear predictors of two species. (e) Species heterogeneity generating covariation over time within each species. (f) Common environmental variance is correlated, gradually declining, over time among species. (g) Within‐species environmental variance is correlated, gradually declining, over time within species. (h) Observation variance adds variance, reducing the overall correlation. (i–k) Sample realizations of community dynamics used in the simulation example. Strengths of density regulation, γ, from 0.05 (i) to 0.5 (k). (i–k) (1) has within‐species variation $\sigma_{e}^{2}$ equal to 0.2, and (i–k) (2) has within‐species variation equal to 0.8. Each simulation consists of 100 species, with two species highlighted in black.

The among‐species random effect, $b_{h}∼N\left(0,\sigma_{h}^{2}I_{S}\right)$ describes the variation in mean abundance among species. Because the mean abundance is proportional to the carrying capacity, the species level random effect can be interpreted as variation in carrying capacity among species. From Equations (3) and (7) we have that the species level random effect can be transformed to variation in density independent growth rate through $\sigma_{r}^{2}=\sigma_{h}^{2}\gamma^{2}$. In Figure 1a we have illustrated the effect of among‐species variation. Each species has a unique carrying capacity sampled from the whole distribution of carrying capacities (horizontal lines [sample] and green square [distribution], respectively, in Figure 1a). The among‐species variation is also called species heterogeneity in the community (Engen & Lande, 1996b), or ecological heterogeneity among species (Lande et al., 2003). From Equation (7) we see that the among‐species variation adds a constant term, $\sigma_{h}^{2}$, to the covariance among‐time points within species (Figure 1e).

The among‐time random effect, $b_{c}∼N\left(0,\sigma_{c}^{2}\rho \right)$, describes variation in mean abundance among‐time points common to all species. The common environmental stochasticity, quantified by the variance $\sigma_{c}^{2}$, describes the environmental effects that have a synchronizing effect on species abundances. The larger $\sigma_{c}^{2}$ is, the more similar abundances will fluctuate over time, as illustrated in Figure 1b. Due to density dependence, the common environmental variance is temporally correlated, generating a non‐negative covariation among species (Figure 1f). From Equations (3) and (7) we have that the temporal level random effect can be transformed to general responses to environmental variation through $\sigma_{E}^{2}=2{\gamma \sigma }_{c}^{2}$.

The species‐temporal random effect, $b_{e}∼N\left(0,\sigma_{e}^{2}I_{S}\;⊗\;\rho \right)$, describes the within‐species variation due to environmental stochasticity. The within‐species variation $\sigma_{e}^{2}$ reduces the synchrony among species within years (please refer to Figure 1c). However, within‐species environmental variance is temporally correlated, determined by *γ* in $\rho_{\mathrm{kl}}=e^{-\gamma |t_{l}-t_{k}|}$, so a small γ gives a larger temporal synchrony in the fluctuations over time (Figure 1c,g). From Equations (3) and (7) we have that the species‐temporal level random effect can be transformed to species‐specific responses to environmental variation through $\sigma_{s}^{2}=2{\gamma \sigma }_{e}^{2}$.

The observation‐level random effect, $b_{o}∼N\left(0,\sigma_{o}^{2}I_{S}\;⊗\;I_{T}\;⊗\;I_{O}\right)$, describes sampling variation due to uncertainty in the measurements or differences in observer skills or any other factor that might result in different abundances of the same species at the same time point. In addition, the observation‐level random effect can account for overdispersion in the Poisson distribution (Browne et al., 2005). However, not all sources of variation can be separated individually, so overdispersion can also include effects such as demographic stochasticity, i.e., variation in individual survival and fecundity, in small local populations (Lande et al., 2003). The extra noise due to observation differences adds a final layer of variation to the observed dynamics (Figure 1d), expressed as an element on the diagonal of the covariance matrix of linear predictors (Figure 1h).

### Simulated example and case studies

In the simulation example, we look at our ability to estimate different variance components and the population dynamic parameters, for different sampling intensities *ν*, and different strengths of density regulation *γ*. The simulations are based on the discrete version of the dynamic model presented in Equation (1) (please refer to the simulation procedure in Appendix S2). We simulate communities with log carrying capacity equal to 10, and strength of density regulation either 0.01, 0.1 or 0.5, so the mean growth rate is accordingly $\mu_{r}=10\gamma$. The within‐species variation $\sigma_{e}^{2}$ is ranging from 0.2,0.4,…,0.8, whereas the among‐species variation is $\sigma_{h}^{2}=1-\sigma_{e}^{2}$, i.e., within‐ and among‐species variations account for 20% to 80% each. The species‐specific response to environmental stochasticity is $\sigma_{s}^{2}=2{\gamma \sigma }_{e}^{2}$, and the ecological heterogeneity among species is $\sigma_{r}^{2}=\gamma^{2}\sigma_{h}^{2}$, expressed as variation in growth rate among species. General effect of environmental stochasticity, $\sigma_{c}^{2}$, is either 0.01, or 0.1, and we have $\sigma_{E}^{2}=2{\gamma \sigma }_{c}^{2}$. The sampling intensity, expressed as proportion of community sampled *ν*, is either 0.01, 0.1, or 0.5. The number of species simulated is 100 that all have the same initial log abundance. The dynamics are simulated for 370 time steps and the first 320 are removed before estimation to ensure that the populations have reached their stationary distributions. Although the assumption of stationary environment is unrealistic, especially over long time periods, the simulations can generate quite different dynamics, as illustrated in Figure 1i–k.

We consider two case studies with data from the open‐access database BioTIME (Dornelas et al., 2018). The first data set is of a fish community in Ria de Aveiro, Portugal. The fish data contained observations from 4 years, and nine locations. We therefore fitted two models, one for modeling temporal dynamics and one for spatial dynamics. The distribution of random effects for the temporal model is

(8) $$b_{\text{fish},\text{time}}=\left[\begin{array}{c} b_{h}\\ b_{e}\\ b_{c} \end{array}\right]∼N\left(\left[\begin{array}{c} 0\\ 0\\ 0 \end{array}\right],\left[\begin{array}{ccc} \sigma_{h}^{2}I_{S} & & \\ & \sigma_{e}^{2}I_{L}\;⊗\;I_{S}\;⊗\;\rho & \\ & & \sigma_{c}^{2}I_{L}\;⊗\;\rho_{c} \end{array}\right]\right),$$

where we estimate the following random effects: the among‐species variation $\sigma_{h}^{2}$, the within‐species variation $\sigma_{e}^{2}$ within locations, and the common environmental variation $\sigma_{c}^{2}$ among locations.

Although the model formulations in Equations (1) and (4) have assumed the same temporal scaling for species‐specific and general response to environmental variation, this might not always be the case, for instance due to competition among species for common resources. To account for this difference, we estimate different correlation parameters for the temporal within‐species and common environmental variation, **ρ** and **ρ**_c_, with inverse temporal scales 1/γ and 1/γ_*c*_, respectively. We have no repeated observations, so we cannot estimate an observation‐level random effect in this example. A similar formulation as in Equation (8) is done for the spatial model (Appendix S3: Equation S1), replacing the species‐specific and general environmental correlation with $e^{-\alpha d\left(z_{l},z_{k}\right)}$ and $e^{-\alpha_{c}d\left(z_{l},z_{k}\right)}$, respectively.

The second data set is of a bat community in the Amazon near Manaus, Brazil. The data set available through BioTIME has 5 years of observations, ranging from 1997 to 2013 at 12 locations. We use additional habitat information from the data set to compare community dynamics for the community as a whole, and split into two data sets, where one is from locations with “continuous forest” habitats, whereas the other is locations from “fragmented interior” habitats. Because there are multiple dates with observations within a single year, we use “date” as repeated observations to illustrate the effect of overdispersion. For general environmental variation we also estimate uncorrelated noise due to sampling units (dates), in which the random effect is defined as $b_{u}∼N\left(0,\sigma_{u}^{2}I_{L}\;⊗\;I_{T}\;⊗\;I_{J}\right)$ and *J* is the number of dates. The exact formulation of random effects for the temporal and spatial models are summarized in Appendix S3: Equations S2 and S3.

When analyzing community dynamics such as the fish and bats data, we estimate several variance and correlation parameters. We can summarize their effect in terms of community similarity by computing the temporal and spatial correlation in relative and mean log abundance. The correlation in relative log abundance consists of all within‐ and among‐species variation components (please refer to Figure 1e,g,h). For example the temporal correlation in relative log abundance within a location in the fish community is $\rho_{x}\left(u\right)=\left(\sigma_{e}^{2}e^{-\gamma u}+\sigma_{h}^{2}\right)/\left(\sigma_{e}^{2}+\sigma_{h}^{2}\right)$. The correlation in mean log abundance is computed using all within‐ and among‐time or location variation components (please refer to Figure 1f), for example the spatial correlation in mean log abundance for the bat community is $\rho_{\bar{X}}\left(v\right)=\sigma_{c}^{2}e^{-\alpha_{c}v}/\left(\sigma_{c}^{2}+\sigma_{u}^{2}\right)$. All correlation functions for the case studies are summarized in Appendix S3: Equations S4–S11.

We assume that for each recorded combination of location, time point and observation replicate, any species without an abundance is a zero in the data set. The models where fitted in *R* (R Core Team, 2021) using the *glmmTMB R*‐package (Brooks et al., 2017) and *DHARMa* R‐package (Hartig, 2021) for residual diagnostics, but note that many GLMM *R*‐packages share the syntax used for demonstration of fitting models here. Parameter uncertainty is estimated using parametric bootstrap (Efron & Tibshirani, 1994) with 1000 replicates in each case. All code used in this paper can be found online at https://doi.org/10.5281/zenodo.6401148 (Solbu et al., 2022), and a detailed guide for the fish data set in Appendix S6.

## RESULTS

### Simulation example

**FIGURE 2:**
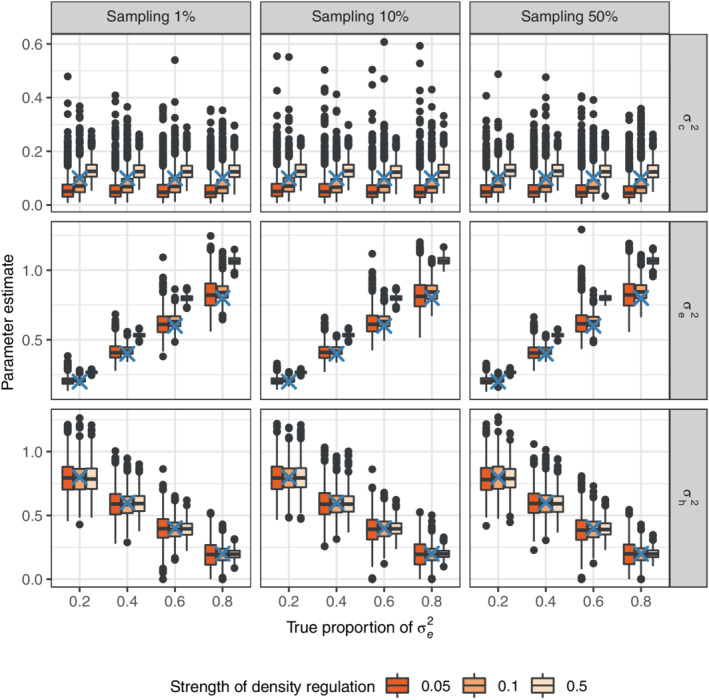
Estimates of common environmental effect (first row), within‐species variation (second row), and among‐species variation (third row), from fitting a generalized linear mixed model to simulated discrete population data. The columns are different sampling intensities, whereas the *x*‐axis is the true proportion of variance due to within‐species variation. The boxplots are point estimates from 1000 simulations, and the blue ‘X’ indicates the true values. Results for common environmental effect $\sigma_{c}^{2}=0.1$ are shown, while $\sigma_{c}^{2}=0.01$ is found in Appendix S5: Figure S3.

**FIGURE 3:**
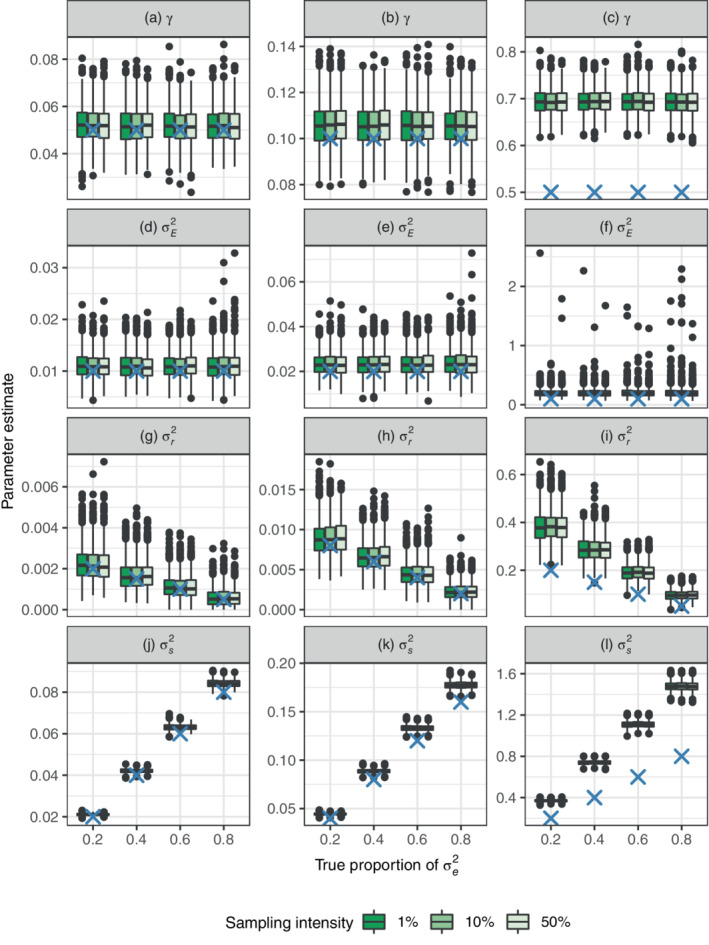
Estimates of strength of density regulation (first row), general response to environmental stochasticity (second row), ecological heterogeneity among species (third row), expressed as variation in growth rate, and species‐specific response to environmental stochasticity (fourth row). The columns have different (true) strengths of density regulation: γ=0.05, 0.1 and 0.5, respectively, whereas the *x*‐axis is the true proportion of variance due to within‐species variation. The boxplots are point estimates from 1000 simulations, and the blue ‘X’ indicates the true values. Results for common environmental effect $\sigma_{c}^{2}=0.1$ are shown, whereas $\sigma_{c}^{2}=0.01$ is found in Appendix S5: Figure S4.

The estimation of the GLMM (Equation 4) on data generated by the discrete version of the population dynamic model (Equation 1) is summarized in Figures 2 and 3. The estimated among‐species variation, $\sigma_{h}^{2}$ is accurate, regardless of sampling intensity and strength of density regulation (Figure 2, third row). The within‐species variation, $\sigma_{e}^{2}$ is overestimated as strength of density regulation increases (Figure 2, second row). The common environmental variance, $\sigma_{c}^{2}$, ranges from under‐ to overestimated as strength of density regulation increases (Figure 2, first row). The results for $\sigma_{c}^{2}=0.01$ were similar, and can be viewed in Appendix S1: Figures S1–S3.

The estimated strength of density regulation is accurate for populations with weak density regulation (Figure 3a,b), and γ is overestimated for strong density regulation (Figure 3c). As a consequence of the estimates of γ, the species‐specific response to environmental stochasticity $\sigma_{s}^{2}$, and ecological heterogeneity among species, expressed as variation in growth rate among species $\sigma_{r}^{2}$, is overestimated for strong density regulation as $\sigma_{s}^{2}=2{\gamma \sigma }_{e}^{2}$ and $\sigma_{r}^{2}=\gamma^{2}\sigma_{h}^{2}$ (Figure 3i,l). Temporal autocorrelation in general environmental effects, γ_*c*_ is overestimated (please refer to Appendix S1: Figure S2), whereas general response to environmental fluctuations $\sigma_{E}^{2}$ is accurate for weak density regulation (Figure 3d,e).

### Fish community

**FIGURE 4:**
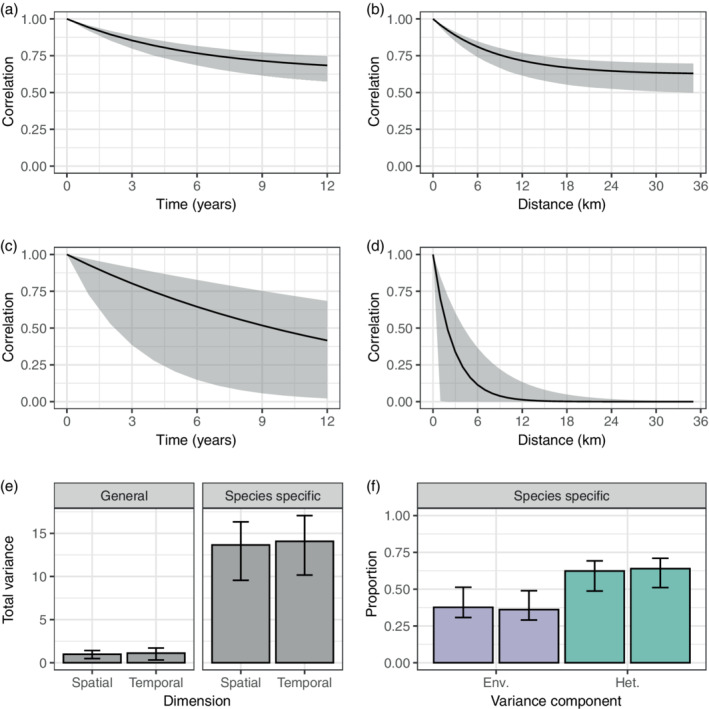
Analysis of fish community data from Ria de Aveiro, Portugal. Temporal (a) and spatial (b) correlations of relative log abundance with gray bands as 95% confidence intervals. Temporal (c) and spatial (d) correlations of mean log abundance with gray bands as 95% confidence intervals. (e) Estimated total variance for mean log abundance (general environmental effects) and relative log abundance (species‐specific effects) with 95% confidence intervals. (f) Proportion of the total variance attributed to temporal environmental variation within‐species ($\sigma_{e}^{2}$, “Env.”), and among‐species variation or ecological heterogeneity, ($\sigma_{h}^{2}$,”Het.”) with 95*%* confidence intervals. For each variance component the columns are from the spatial (left) and temporal (right) models. All confidence intervals are based on 1000 bootstrap replicates.

In the fish community case study, the estimated total variance of the species abundance distribution, the variance in relative log abundance (Equation 2), is 14.1 based on the temporal GLMM (Equation 4, Figure 4e; Appendix S4: Table S1). Our approach estimates that 64% of this variation is due to ecological heterogeneity among species, whereas the remaining proportion is attributed to species‐specific responses to environmental stochasticity (Figure 4f). Variation in the mean log abundance, due to general environmental stochasticity, is roughly one for both the temporal and spatial model. The estimated strength of density regulation is $\hat{\gamma }=0.17\;{\text{year}}^{-1}$ (the hat symbol ‘$\hat{\;}$’ indicates estimate), so the corresponding temporal scale $\hat{1/\gamma }$ is 5.8 years. In Figure 4c we see how the uncertainty in temporal correlation for the general environment, the dynamic of mean log abundance, is much larger than the species‐specific in Figure 4a, which is the dynamic of relative log abundance. The difference in uncertainty is because the general environment is estimated with only four time points over nine levels (locations), whereas the species‐specific has four time points over 648 levels (species and locations).

### Bat community

**FIGURE 5:**
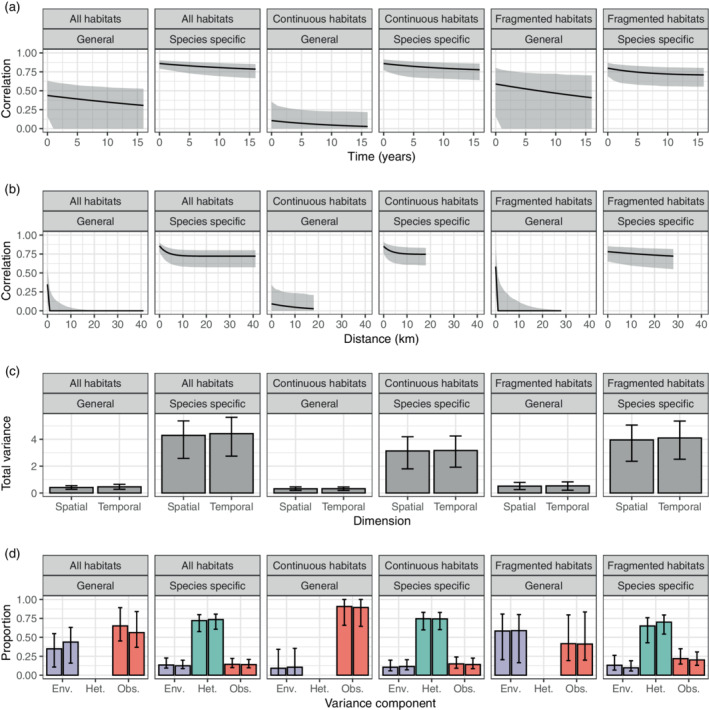
Analysis of bat community data from Manaus, Brazil. Temporal (a) and spatial (b) correlation in “General” mean log abundance and “Species specific” relative log abundance, with gray bands as 95% confidence intervals. (c) Estimated total variance for mean log abundance (general environmental effects) and relative log abundance (species‐specific effects) with 95% confidence intervals. (d) Proportion of the total variance attributed to temporal environmental variation within‐species $\sigma_{e}^{2}$, among‐species variation or ecological heterogeneity $\sigma_{h}^{2}$, and observation‐level random effects or overdispersion $\sigma_{o}^{2}$ with 95% confidence intervals for species‐specific effects. For the general effects we have environmental variation ($\sigma_{c}^{2}$, “Env.”) and uncorrelated random effects ($\sigma_{u}^{2}$, “Obs.”). For each variance component the columns are from the spatial (left) and temporal (right) models. All confidence intervals are based on 1000 bootstrap replicates.

In the bat community case study, the among‐species variation, or ecological heterogeneity among species, accounts for 74% of the total variation in the species abundance distribution when analyzing all habitats together (Figure 5d and Appendix S4: Table S2). Species‐specific response to environmental stochasticity accounts for another 12% of the variance, whereas the final 14% is due to overdispersion. The large amount of overdispersion is not surprising because we have used samples from different dates as “repeated observations” of the community. Seasonal variation in species composition will affect this estimate, in addition to demographic stochasticity and intraspecific aggregation (Engen et al., 2002; Lande et al., 2003). The partitioning of the variance is similar for the “continuous” and “fragmented” habitats analyzed separately, but “continuous” habitats have a smaller total variance, 3.17 compared with 4.10 in “fragmented” habitats. Although the proportions of variance are almost identical (74‐12‐14% “continuous,” 70‐10‐20% “fragmented”), the spatial and temporal scaling differ considerably. The “continuous” habitats have a much weaker strength of density regulation $\hat{\gamma }\;=\;0.08\;{\text{year}}^{-1}$, half that of the “fragmented” habitats $\hat{\gamma }\;=\;0.16\;{\text{year}}^{-1}$. This means that the temporal scaling, also known as the mean return time to equilibrium, is twice as long for “continuous” habitats compared with fragmented ones. The spatial scale is shorter for “continuous” habitats than “fragmented” ones, with $\hat{1/\alpha }\;=\;3.4\;\mathrm{km}$ and $\hat{1/\alpha }\;=\;46\;\mathrm{km}$, respectively. One interpretation of this difference is that the composition of species has a higher rate of spatial turnover for “continuous” compared with “fragmented” habitats. A consequence of splitting up the data set into two smaller ones, is that the uncertainty, particularly in the population dynamic parameters, increases considerably. However, it serves to showcase how communities with different dynamics can generate similar variance compositions.

## DISCUSSION

In this paper we showed how the dynamic species abundance distribution can be fitted using the GLMM framework. The main benefit is that we can use standard statistical tools to analyze the community dynamics more directly compared with previous approaches (Engen et al., 2002; Grøtan & Engen, 2008). With the accessibility and speed of analyzing dynamic species abundance distributions as GLMMs, population dynamics has the potential to affect the ability of ecologists to understand and evaluate different sources of variation with much greater flexibility than before.

Comparing the results from simulations of discrete population dynamic models to estimates from the GLMM illustrates how the modeling approach, i.e., the approximation of dynamics by a continuous time process, is more accurate for weaker density regulation than strong density regulation. This is due to the more volatile fluctuations that strong density regulation generates (Figure 1k), which is not accurately approximated by the continuous time population model (Equation 1). But if the dynamics are slower than a mean return time of two time units, meaning that γ<0.5, the estimation results are more reliable (Figure 1i,j). Therefore, if the estimated strength of density regulation is higher than, say 0.5, parameter estimates should be interpreted with caution. One solution is to model the relative log abundance over shorter time intervals, for example monthly instead of yearly, if the data needed to do this are available. Among‐species variation, expressed as variation in carrying capacity, can be accurately estimated even when the strength of density regulation is high (Figure 2). The general environmental effects, describing dynamics in mean log abundance, have fewer levels than the species‐specific effects, describing dynamics of relative abundance, and are therefore harder to estimate with high accuracy (Figures 4c,d and 5a,b, Bolker et al., 2009; Harrison et al., 2018; Silk et al., 2020). It is, however, important to account for the variation in mean log abundance among locations and time points by either fixed or random effects, otherwise it will bias the estimates of variation in relative log abundance.

The modeling approach in this paper makes it possible for ecologists to estimate variance components of dynamic species abundance distributions that have direct connections to population dynamics. For instance, two communities can have the same total variance, but different proportions attributed to environmental variance and species heterogeneity, which in turn will give potentially very different conclusions regarding, for example, the probability of extinction for individual species, which increases with environmental stochasticity (Lande et al., 2003). Similarly, two communities can have the same magnitude and composition of variance, but different mean return times to equilibrium, which affect for instance how species respond to changes in carrying capacity due to, for example, changes in habitat or climate. In the bat community, where the variance composition was almost identical for “continuous” and “fragmented” habitats, we estimated a much shorter temporal scaling for “fragmented” communities compared with “continuous” habitats. This can be interpreted as higher pressure through density regulation due to smaller habitats in fragmented areas, but the uncertainty makes it hard to conclude whether this is a true pattern.

For the fish community case study, the main cause of variation in community composition was species heterogeneity, expressed as variance in log carrying capacity, with 64% of the total variance, whereas 36% is attributable to environmental fluctuations. The results are consistent with other studies, although analyzed over different time ranges. Rebelo (1992) studied monthly variation from August 1987 to July 1988 in species abundance at 10 locations in the area and found that eight out of 55 fish species recorded accounted for 80% of the total abundance and six species were recorded at all time points. The findings of Rebelo (1992) suggest that the monthly dynamics of the fish community is generated by ecological heterogeneity, as some species are continuously abundant over the study period, whereas other less abundant species generate the species turnover in the community.

The correlation structure of the fitted dynamical species abundance model can be used to understand community turnover, and used as a measure of temporal β‐diversity (Anderson et al., 2011; Magurran et al., 2019). If the correlation in relative log abundance rapidly approaches a limiting value as the interval between observations increases, it could indicate a fast turnover rate in the community. If this is the case, the strength of density regulation is an estimate of the rate of change in turnover along a temporal gradient (Anderson et al., 2011). Similarly, assuming the same exponential decay for spatial correlation, the parameter *α* expresses the turnover along a spatial gradient. For the fish community, the synchrony due to species‐specific responses to environmental fluctuations decreases by a fraction $e^{-1}$ after roughly 8.6 km or 5.8 years, respectively. This indicates little spatial synchrony due to environmental fluctuations, as locations are mostly further away from each other, supporting previous reports of considerable spatial variation, whereas temporal diversity was more stable (Pombo et al., 2005, 2007).

Defined as a GLMM, the dynamic species abundance distribution can also be formulated as a joint species distribution model (JSDM; Warton et al., 2015). The main difference to other JSDMs is that dynamic species abundance distributions are used to model community dynamics, such as temporal correlations, whereas JSDMs are often used to analyze correlation between species to understand the complexity of species co‐occurrence patterns (Ovaskainen et al., 2016; Pollock et al., 2014; Tobler et al., 2019). For JSDMs of species interactions in species rich communities, the solution to reduce the number of parameters is to apply dimension reduction techniques to the residual covariance matrix, known as generalized linear latent variable modeling (Hui et al., 2015; Niku et al., 2019; Warton et al., 2015). However, at the community level, the impact of species interactions is small compared with environmental fluctuations (Mutshinda et al., 2009, 2011). There are more complex models that incorporate density dependent dynamics, interactions and spatio‐temporal variation to analyze joint dynamic species distribution models (Thorson et al., 2016), but the accessibility of GLMM analysis makes the connections presented here a valuable addition to multispecies ecological modeling.

The analysis of ecological communities with dynamic species abundance distributions draws upon themes that have been of interest to ecologists for over a century (McGill, 2011; Rosenzweig, 1995). By using the approach described in this paper, more information can be extracted by partitioning the residual variance into components so that each are associated with population processes and variation in space and time. Obtaining the population dynamic parameters that describe how the populations will behave over time is key to understanding how communities will react to environmental changes (Shoemaker et al., 2020). This is particularly important given the large environmental changes occurring globally, which both affect the spatial heterogeneity through habitat fragmentation and deterioration, and temporal heterogeneity through a more unpredictable climate. Knowledge on how communities respond to such changes is therefore pivotal to understand and act to reduce the ongoing biodiversity crisis (Johnson et al., 2017). With the implementation of dynamics species abundance distributions such as GLMMs, there are more opportunities to analyze a wide array of different data sets within this framework, which can provide greater understanding of community dynamics in a changing world.

## CONFLICT OF INTEREST

The authors declare no conflict of interest.

## Supporting information


Appendix S1

Appendix S2

Appendix S3

Appendix S4

Appendix S5

Appendix S6

## Data Availability

Code is provided via the following link: https://doi.org/10.5281/zenodo.6401148 (Solbu et al., 2022). Data sets utilized for this research are found in Dornelas et al. (2018) and can be downloaded at https://doi.org/10.5281/zenodo.5026943 or at https://biotime.st-andrews.ac.uk/selectStudy.php?study=467 (fish) and https://biotime.st-andrews.ac.uk/selectStudy.php?study=516 (bats).
