# Supplementary material for: Analyzing dynamic species abundance distributions using generalized linear mixed models
Source: Ecology. 2022 Jun 23;103(9):e3742. doi: 10.1002/ecy.3742 (PMC9541646; doi:10.1002/ecy.3742)
Supplement: Supplementary file 1 — Appendix S1 [file ECY-103-e3742-s005.pdf]

Supporting Information for "Analyzing dynamic species abundance distributions using generalized linear mixed models" in Ecology by Erik Blystad Solbu, Bert van der Veen, Ivar Herfindal and Knut Anders Hovstad.

Appendix S1: Summary of notation

Notation in "Dynamic species abundance distributions"	
i	index of species, $1, \ldots, S$
S	total number of species
X_i	log abundance of species i
r_i	density-independent growth rate of species i
μ_r	mean value of r_i among species, following a normal distribution
σ_r^2	variance of r_i among species, following a normal distribution
γ	strength of density regulation (assumed same for all species)
σ_E^2	general response to environmental variability
σ_s^2	species-specific response to environmental variability
t	index of time $t_1, \ldots, t_T$
T	total number of time points
$dB_i(t)$	Brownian motions indexed by species i and time t
$dE(t)$	Brownian motions indexed by time t
$M(dX) = a + bX$	infinitesimal mean, expected change over a very short time interval, assumed to be a linear function
$V(dX) = c$	infinitesimal variance, expected variation over a very short time interval, assumed to be constant
$m_X = \frac{a}{b}$	stationary mean of the diffusion process X
$\sigma_X^2 = \frac{c}{2b}$	stationary variance of the diffusion process X
$\bar{X} = \frac{1}{S}\sum_{i=1}^{S} X_i$	mean log abundance
$x_i = X_i - \bar{X}$	relative log abundance
$\bar{r} = \frac{1}{S}\sum_{i=1}^{S} r_i$	sample mean growth rate

Notation in "GLMM formulation"	
z	index of locations $z_1, \dots, z_L$
L	total number of locations
j	index of observation replicate $1, \dots, J$
J	total number of replicates
$y_{ij}(t, z)$	observation replicate j of the abundance of species i at time t and location z
$\mu_{ij}(t, z)$	mean value of $y_{ij}(t, z)$
$\eta_{ij}(t, z) = \log[\mu_{ij}(t, z)]$	linear predictor of the log-transformed $\mu_{ij}(t, z)$
$N = S \cdot T \cdot L \cdot J$	total number of observations
$\boldsymbol{\eta}$	vector of dimension $N \times 1$ of linear predictors
$\boldsymbol{X}$	design-matrix for fixed effects. In all cases here we assume $\boldsymbol{X} = \mathbf{1}_N$
$\boldsymbol{\beta}$	vector of fixed effects. In all cases we assume $\boldsymbol{\beta}$ is scalar β_0
$\boldsymbol{Z}$	design-matrix for random effects
$\boldsymbol{b}$	vector of random effects
$\boldsymbol{Z}_h$	design-matrix for random effects due to among-species variation
$\boldsymbol{Z}_e$	design-matrix for random effects due to within-species variation
$\boldsymbol{Z}_c$	design-matrix for random effects due to among-time points variation
$\boldsymbol{Z}_o$	design-matrix for random effects due to among-observation variation
$\boldsymbol{b}_h = [h_1, \dots, h_S]'$	vector of random effects due to among-species variation, i.e. one random element h_i for each species
$\boldsymbol{b}_e = [e_1(1), \dots, e_S(T)]'$	vector of random effects due to within-species variation, i.e. one random element $e_i(t)$ for each species at each time point
$\boldsymbol{b}_c = [c(1), \dots, c(T)]'$	vector of random effects due to among-time points variation, i.e. one random element $c(t)$ for each time point
$\boldsymbol{b}_o = [\varepsilon_{11}(1), \dots, \varepsilon_{SJ}(T)]'$	vector of random effects due to variation among observations, i.e. one random element $\varepsilon_{ij}(t)$ for each observation
$\boldsymbol{I}_S, \boldsymbol{I}_T, \boldsymbol{I}_J$	identity matrix (1s on the diagonal, 0s otherwise) of order S, T, or J (rows and columns), depending on the subscript of $\boldsymbol{I}$
$\mathbf{1}_S, \mathbf{1}_T, \mathbf{1}_J$	vector of 1s of dimension $S \times 1$, $T \times 1$, or $J \times 1$, depending on the subscript of $\mathbf{1}$
σ_h^2	variance of among-species random effects $\boldsymbol{b}_h$
σ_e^2	variance of within-species random effects $\boldsymbol{b}_e$
σ_c^2	variance of among-time points random effects $\boldsymbol{b}_c$
σ_o^2	variance of observation level random effects $\boldsymbol{b}_o$
$\boldsymbol{\rho}$	$T \times T$ symmetric matrix with elements $\rho_{kl} = e^{-\gamma\lvert t_l - t_k \rvert}$, for $k, l \in 1, \dots, T$
$\boldsymbol{\rho}$	$L \times L$ symmetric matrix with elements $\rho_{kl} = e^{-\alpha d(z_l, z_k)}$, for $k, l \in 1, \dots, L$

Notation in "Interpretation of intercept and random effects"	
ν	sampling intensity
$\ln K$	log carrying capacity
$\tilde{\beta}_0$	estimated intercept, confounded by sampling intensity

Notation in "Simulation example and case studies"	
$1/\gamma_c$	temporal scaling in general response to environmental variation
$1/\alpha_c$	spatial scaling in general response to environmental variation
$\boldsymbol{b}_u = [u_1(1,1), \ldots, u_J(T,L)]'$	vector of random effects due to variation among observation units
σ_u^2	uncorrelated variation in mean log abundance
$\rho_x(u)$	temporal correlation in relative log abundance within locations
$\rho_{\bar{X}}(u)$	temporal correlation in mean log abundance within locations
