# Supplementary material for: Analyzing dynamic species abundance distributions using generalized linear mixed models
Source: Ecology. 2022 Jun 23;103(9):e3742. doi: 10.1002/ecy.3742 (PMC9541646; doi:10.1002/ecy.3742)
Supplement: Supplementary file 2 — Appendix S2 [file ECY-103-e3742-s006.pdf]

Supporting Information for "Analyzing dynamic species abundance distributions using generalized linear mixed models" in Ecology by Erik Blystad Solbu, Bert van der Veen, Ivar Herfindal and Knut Anders Hovstad.

Appendix S2: Simulation of stochastic population dynamics

Input parameters: S: number of species
T: number of time points
γ: strength of density regulation
σ_s^2: species-specific response to environmental variation
σ_r^2: among-species variation in growth rate
σ_E^2: general response to environmental variation
r_0: mean growth rate among species

- sample S growth rates from $N(r_0, \sigma_r^2)$, assigned to $\boldsymbol{r}$
- set initial abundance to all species equal to $\boldsymbol{x}[0] = r_0/\gamma$
- sample T general responses to environmental variation from $N(0, \sigma_E^2)$, assigned to $\boldsymbol{c}$
- for t from 1 to T, do:
  - sample S species specific responses to environmental variation from $N(0, \sigma_s^2)$, assigned to $\boldsymbol{s}$
  - compute $\boldsymbol{x}[t] = \boldsymbol{x}[t-1] + \boldsymbol{r} - \gamma\boldsymbol{x}[t-1] + \boldsymbol{c}[t] + \boldsymbol{s}$
  - if any element of $\boldsymbol{x}[t] < 0$, set that element equal to zero
