# Supplementary material for: Analyzing dynamic species abundance distributions using generalized linear mixed models
Source: Ecology. 2022 Jun 23;103(9):e3742. doi: 10.1002/ecy.3742 (PMC9541646; doi:10.1002/ecy.3742)
Supplement: Supplementary file 3 — Appendix S3 [file ECY-103-e3742-s001.pdf]

Supporting Information for "Analyzing dynamic species abundance distributions using generalized linear mixed models" in Ecology by Erik Blystad Solbu, Bert van der Veen, Ivar Herfindal and Knut Anders Hovstad.

Appendix S3: Additional distributions of random effects and correlation functions in case studies

The distribution of random effects for the spatial model for the fish data is

$$\boldsymbol{b}_{\text{fish,space}} = \begin{bmatrix} \boldsymbol{b}_h \\ \boldsymbol{b}_e \\ \boldsymbol{b}_c \end{bmatrix} \sim N\left(\begin{bmatrix} \mathbf{0} \\ \mathbf{0} \\ \mathbf{0} \end{bmatrix}, \begin{bmatrix} \sigma_h^2 \boldsymbol{I}_S & & \\ & \sigma_e^2 \boldsymbol{I}_T \otimes \boldsymbol{I}_S \otimes \boldsymbol{\rho} & \\ & & \sigma_c^2 \boldsymbol{I}_T \otimes \boldsymbol{\rho}_c \end{bmatrix} \right). \qquad \text{(Eq. S1)}$$

The distribution of random effects for the temporal model for the bats data is

$$\boldsymbol{b}_{\text{bats,time}} = \begin{cases} \boldsymbol{b}_h \sim N\left(\mathbf{0}, \sigma_h^2 \boldsymbol{I}_S\right) \\ \boldsymbol{b}_o \sim N\left(\mathbf{0}, \sigma_o^2 \boldsymbol{I}_L \otimes \boldsymbol{I}_S \otimes \boldsymbol{I}_T \otimes \boldsymbol{I}_J\right) \\ \boldsymbol{b}_e \sim N\left(\mathbf{0}, \sigma_e^2 \boldsymbol{I}_L \otimes \boldsymbol{I}_S \otimes \boldsymbol{\rho}\right) \\ \boldsymbol{b}_c \sim N\left(\mathbf{0}, \sigma_c^2 \boldsymbol{I}_L \otimes \boldsymbol{\rho}_c\right) \\ \boldsymbol{b}_u \sim N\left(\mathbf{0}, \sigma_u^2 \boldsymbol{I}_L \otimes \boldsymbol{I}_T \otimes \boldsymbol{I}_J\right) \end{cases} . \qquad \text{(Eq. S2)}$$

The distribution of random effects for the spatial model for the bats data is

$$\boldsymbol{b}_{\text{bats,space}} = \begin{cases} \boldsymbol{b}_h \sim N\left(\mathbf{0}, \sigma_h^2 \boldsymbol{I}_S\right) \\ \boldsymbol{b}_o \sim N\left(\mathbf{0}, \sigma_o^2 \boldsymbol{I}_L \otimes \boldsymbol{I}_S \otimes \boldsymbol{I}_T \otimes \boldsymbol{I}_J\right) \\ \boldsymbol{b}_e \sim N\left(\mathbf{0}, \sigma_e^2 \boldsymbol{I}_T \otimes \boldsymbol{I}_S \otimes \boldsymbol{\rho}\right) \\ \boldsymbol{b}_c \sim N\left(\mathbf{0}, \sigma_c^2 \boldsymbol{I}_T \otimes \boldsymbol{\rho}_c\right) \\ \boldsymbol{b}_u \sim N\left(\mathbf{0}, \sigma_u^2 \boldsymbol{I}_L \otimes \boldsymbol{I}_T \otimes \boldsymbol{I}_J\right) \end{cases} . \qquad \text{(Eq. S3)}$$

Correlations for fish analysis:

$$\text{Temporal correlation in relative log abundance:} \quad \rho_x(u) = \frac{\sigma_e^2 e^{-\gamma u} + \sigma_h^2}{\sigma_e^2 + \sigma_h^2} \qquad \text{(Eq. S4)}$$

$$\text{Spatial correlation in relative log abundance:} \quad \rho_x(v) = \frac{\sigma_e^2 e^{-\alpha v} + \sigma_h^2}{\sigma_e^2 + \sigma_h^2}, \qquad \text{(Eq. S5)}$$

$$\text{Temporal correlation in mean log abundance:} \quad \rho_{\bar{X}}(u) = e^{-\gamma_c u}, \qquad \text{(Eq. S6)}$$

$$\text{Spatial correlation in mean log abundance:} \quad \rho_{\bar{X}}(v) = e^{-\alpha_c v}, \qquad \text{(Eq. S7)}$$

Correlations for bats analysis:

$$\text{Temporal correlation in relative log abundance:} \quad \rho_x(u) = \frac{\sigma_e^2 e^{-\gamma u} + \sigma_h^2}{\sigma_e^2 + \sigma_h^2 + \sigma_o^2} \qquad \text{(Eq. S8)}$$

$$\text{Spatial correlation in relative log abundance:} \quad \rho_x(v) = \frac{\sigma_e^2 e^{-\alpha v} + \sigma_h^2}{\sigma_e^2 + \sigma_h^2 + \sigma_o^2}, \qquad \text{(Eq. S9)}$$

$$\text{Temporal correlation in mean log abundance:} \quad \rho_{\bar{X}}(u) = \frac{\sigma_c^2 e^{-\gamma_c u}}{\sigma_c^2 + \sigma_u^2}, \qquad \text{(Eq. S10)}$$

$$\text{Spatial correlation in mean log abundance:} \quad \rho_{\bar{X}}(v) = \frac{\sigma_c^2 e^{-\alpha_c v}}{\sigma_c^2 + \sigma_u^2}, \qquad \text{(Eq. S11)}$$
