# Supplementary material for: Analyzing dynamic species abundance distributions using generalized linear mixed models
Source: Ecology. 2022 Jun 23;103(9):e3742. doi: 10.1002/ecy.3742 (PMC9541646; doi:10.1002/ecy.3742)
Supplement: Supplementary file 4 — Appendix S4 [file ECY-103-e3742-s004.pdf]

Supporting Information for "Analyzing dynamic species abundance distributions using generalized linear mixed models" in Ecology by Erik Blystad Solbu, Bert van der Veen, Ivar Herfindal and Knut Anders Hovstad.

# Appendix S4: Tables of results

| | Parameter | Estimate | 95% CI |
|---|---|---|---|
| Within samples | Among species - ecological heterogeneity, $\sigma_h^2$ | 9.00 | (5.35, 11.91) |
| | | *8.51* | *(4.69, 11.13)* |
| | Within species - environmental effects, $\sigma_e^2$ | 5.08 | (4.29, 5.63) |
| | | *5.14* | *(4.31, 5.73)* |
| | Temporal scale, $1/\gamma$ | 5.78 | (4.72, 7.25) |
| | Spatial scale, $1/\alpha$ | *8.57* | *(7.17, 10.46)* |
| Among samples | General environment, $\sigma_c^2$ | 1.11 | (0.32, 1.71) |
| | | *0.99* | *(0.48, 1.41)* |
| | Temporal scale, $1/\gamma_c$ | 13.67 | (3.14, 31.6) |
| | Spatial scale, $1/\alpha_c$ | *2.76* | *(0.18, 5.95)* |
| Dynamic parameters | Strength of density regulation, $\gamma$ | 0.17 | (0.14, 0.21) |
| | Species-specific response to environment, $\sigma_s^2$ | 1.76 | (1.39, 2.02) |
| | Variation in growth rate among species, $\sigma_r^2$ | 0.27 | (0.13, 0.41) |

Table S1: Estimates and confidence intervals (CI) of variance components, scaling and dynamic parameters for the fish community data from Ria de Aveiro, Portugal. The confidence intervals are based on 1000 bootstrap replicates. Results in italics are from the spatial model.

| | All | | Fragmented | | Continuous | |
|---|---|---|---|---|---|---|
| Parameter | Estimate | 95% CI | Estimate | 95% CI | Estimate | 95% CI |
| Within samples | | | | | | |
| Among species - ecological heterogeneity, $\sigma_h^2$ | 3.25 | (1.66, 4.56) | 2.87 | (1.39, 4.19) | 2.35 | (1.17, 3.48) |
| | *3.10* | *(1.49, 4.28)* | *2.57* | *(1.05, 3.75)* | *2.34* | *(1.1, 3.43)* |
| Within species - environmental effects, $\sigma_e^2$ | 0.55 | (0.4, 0.66) | 0.40 | (0.22, 0.59) | 0.37 | (0.22, 0.51) |
| | *0.58* | *(0.4, 0.72)* | *0.52* | *(0.25, 0.82)* | *0.33* | *(0.18, 0.48)* |
| Observation level - overdispersion, $\sigma_o^2$ | 0.62 | (0.44, 0.67) | 0.83 | (0.55, 0.96) | 0.45 | (0.28, 0.53) |
| | *0.62* | *(0.44, 0.67)* | *0.86* | *(0.58, 1.01)* | *0.47* | *(0.29, 0.58)* |
| Temporal scale, $1/\gamma$ | 17.82 | (8.23, 33.98) | 6.35 | (0.73, 25.49) | 13.17 | (1.19, 41.95) |
| Spatial scale, $1/\alpha$ | *3.29* | *(1.87, 5.8)* | *46.51* | *(15.15, 480.55)* | *3.38* | *(1.15, 11.12)* |
| Among samples | | | | | | |
| General environment, $\sigma_c^2$ | 0.20 | (0.05, 0.38) | 0.31 | (0.04, 0.62) | 0.03 | (0, 0.13) |
| | *0.14* | *(0.04, 0.26)* | *0.30* | *(0.07, 0.56)* | *0.03* | *(0, 0.12)* |
| Uncorrelated noise, $\sigma_u^2$ | 0.26 | (0.17, 0.34) | 0.22 | (0.09, 0.34) | 0.28 | (0.16, 0.4) |
| | *0.27* | *(0.17, 0.37)* | *0.21* | *(0.09, 0.36)* | *0.29* | *(0.16, 0.4)* |
| Temporal scale, $1/\gamma_c$ | 44.98 | (0.4, $\infty$) | 43.62 | (0.07, $\infty$) | 12.24 | (0.05, $\infty$) |
| Spatial scale, $1/\alpha_c$ | *0.04* | *(0.04, 5.45)* | *0.02* | *(0.01, 7.48)* | *14.944* | *(0.04, $\infty$)* |
| Dynamic parameters | | | | | | |
| Strength of density regulation, $\gamma$ | 0.06 | (0.03, 0.12) | 0.16 | (0.04, 1.37) | 0.08 | (0.02, 0.84) |
| Species-specific response to environment, $\sigma_s^2$ | 0.06 | (0.03, 0.13) | 0.13 | (0.02, 1.11) | 0.06 | (0.01, 0.59) |
| Variation in growth rate among species, $\sigma_r^2$ | 0.01 | (0.002, 0.04) | 0.07 | (0.004, 4.49) | 0.01 | (0.001, 1.33) |

Table S2: Estimates and confidence intervals (CI) of variance components, scaling and dynamic parameters for the bat community data from Manaus, Brazil. The confidence intervals are based on 1000 bootstrap replicates. Results in italics are from the spatial model.
