# Supplementary material for: Analyzing dynamic species abundance distributions using generalized linear mixed models
Source: Ecology. 2022 Jun 23;103(9):e3742. doi: 10.1002/ecy.3742 (PMC9541646; doi:10.1002/ecy.3742)
Supplement: Supplementary file 5 — Appendix S5 [file ECY-103-e3742-s003.pdf]

Supporting Information for "Analyzing dynamic species abundance distributions using generalized linear mixed models" in Ecology by Erik Blystad Solbu, Bert van der Veen, Ivar Herfindal and Knut Anders Hovstad.

Appendix S5: Additional figures

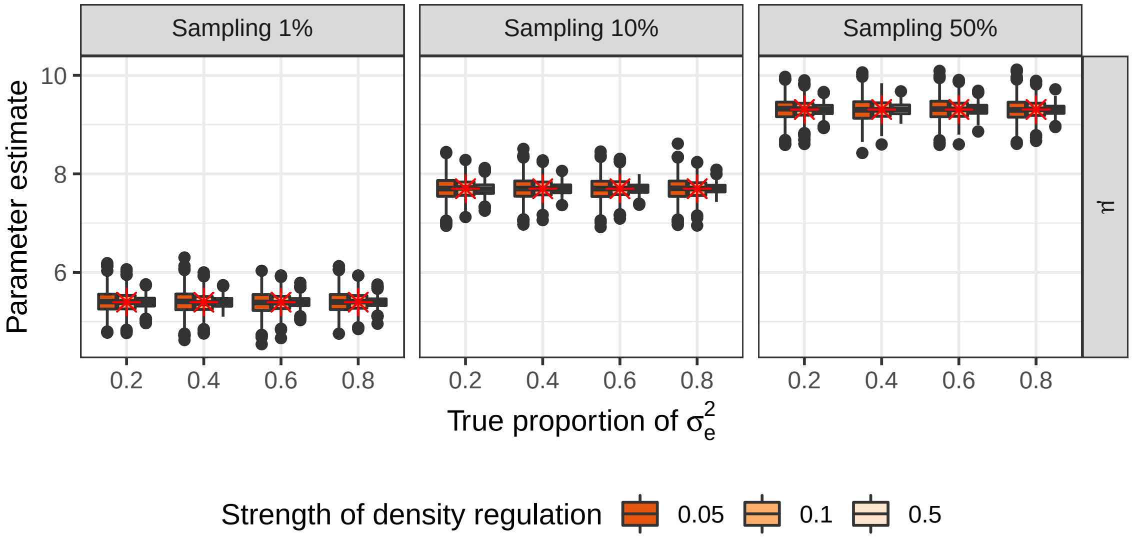

Figure S1: Estimates of mean log abundance (first row) from fitting a GLMM to simulated discrete population data. The columns are different sampling intensities, while the x-axis is the true proportion of variance due to within-species variation. The box-plots are point estimates from 1000 simulations, and the red asterisks indicate the true values. Results for common environmental effect $\sigma_c^2 = 0.1$ shown.

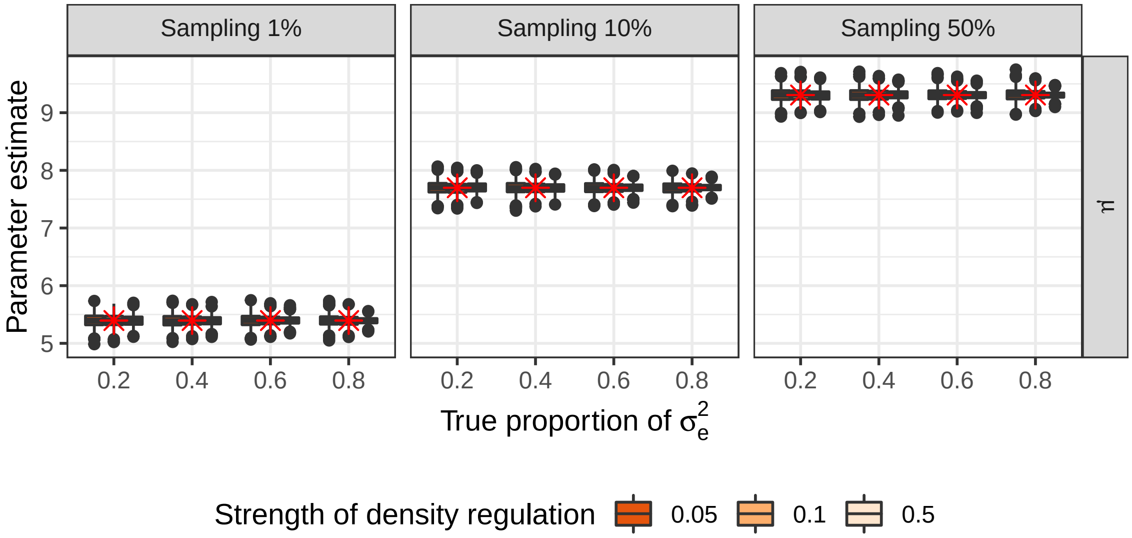

Figure S2: Estimates of mean log abundance (first row) from fitting a GLMM to simulated discrete population data. The columns are different sampling intensities, while the x-axis is the true proportion of variance due to within-species variation. The box-plots are point estimates from 1000 simulations, and the red asterisks indicate the true values. Results for common environmental effect $\sigma_c^2 = 0.01$ shown.

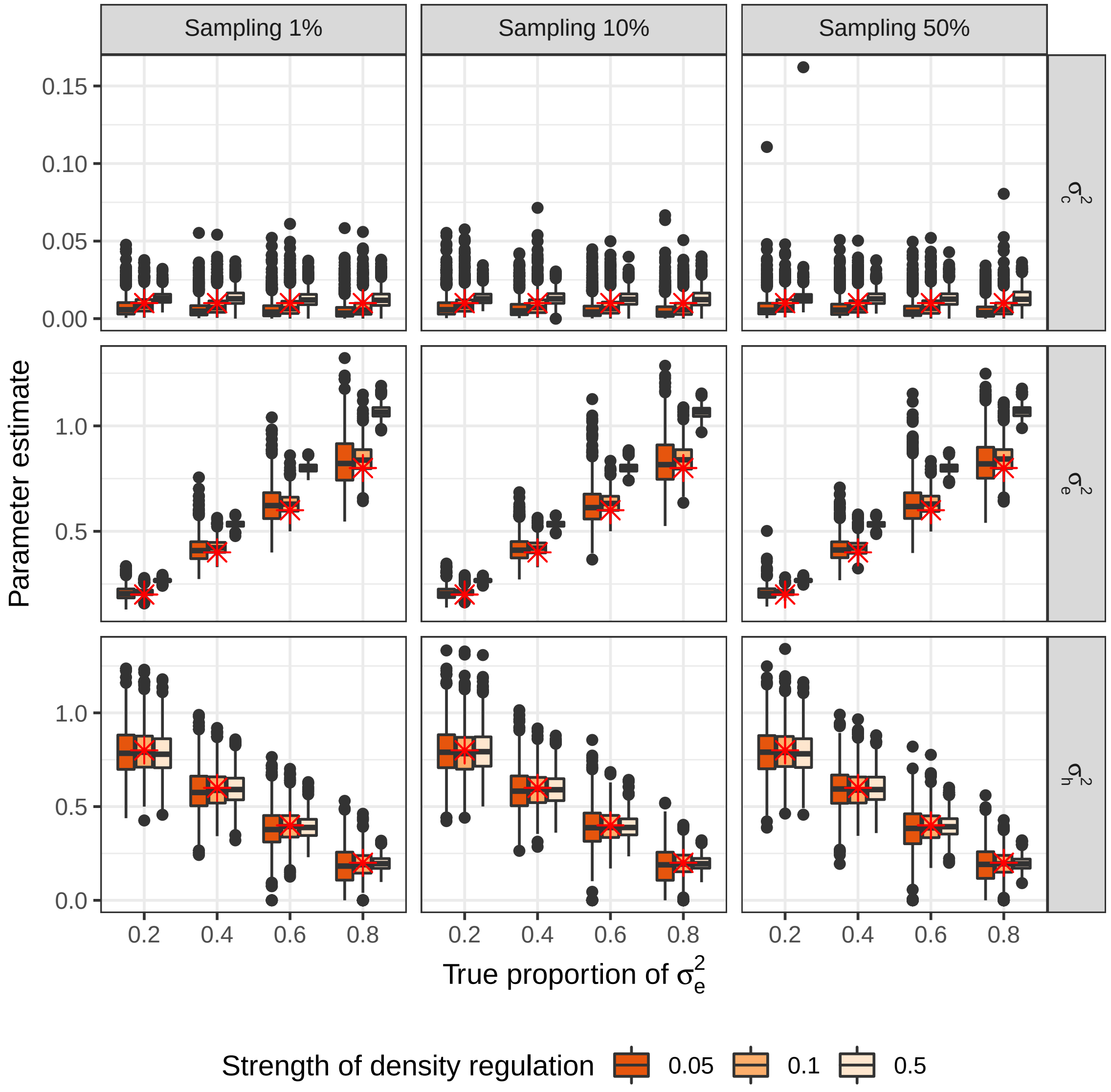

Figure S3: Estimates of mean log abundance (first row), common environmental effect (second row), within-species variation (third row), and among-species variation (fourth row), from fitting a GLMM to simulated discrete population data. The columns are different sampling intensities, while the x-axis is the true proportion of variance due to within-species variation. The box-plots are point estimates from 1000 simulations, and the red asterisks indicate the true values. Results for common environmental effect $\sigma_c^2 = 0.01$ shown.

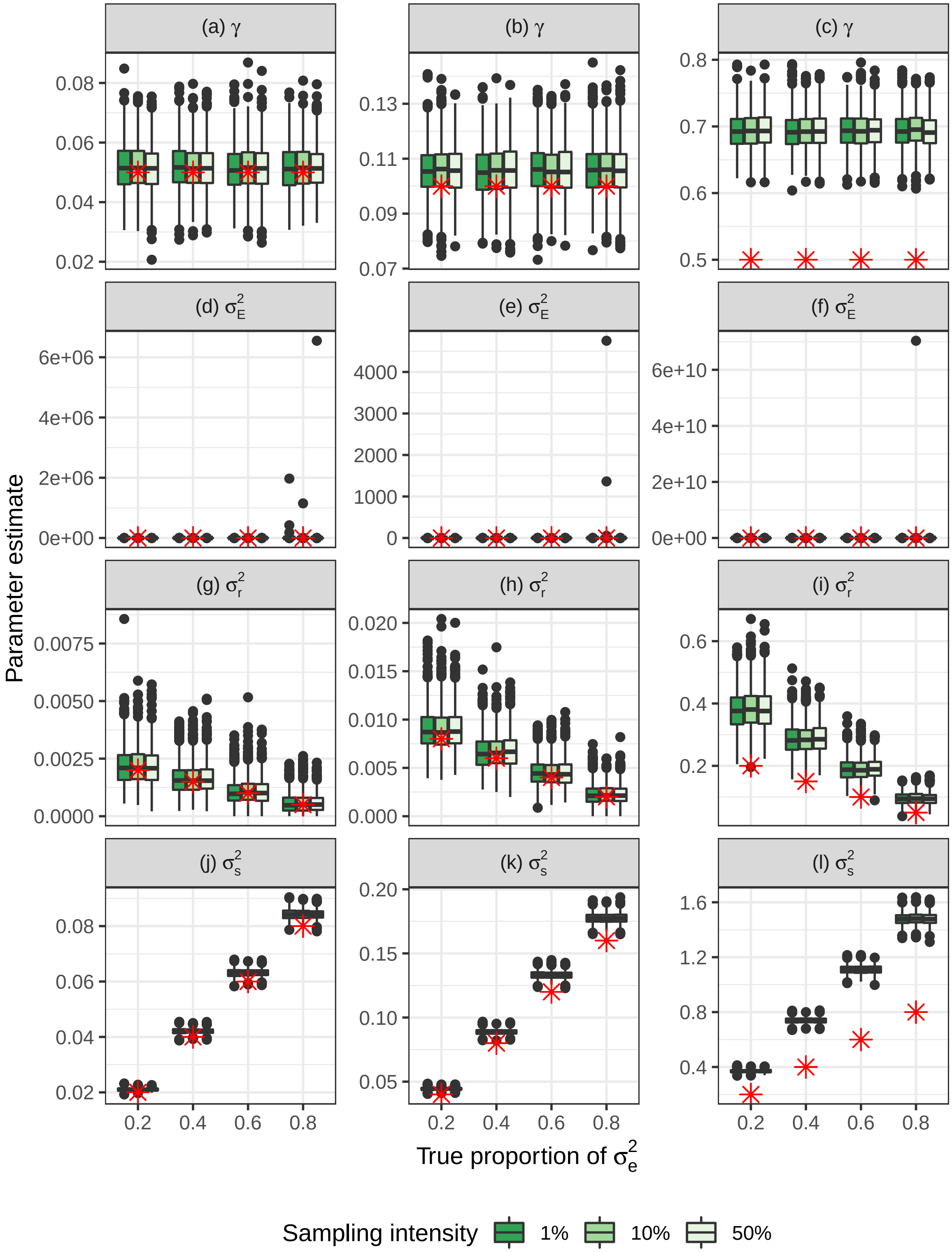

Figure S4: Estimates of strength of density regulation (first row), general response to environmental stochasticity (second row), ecological heterogeneity among species (third row), expressed as variation in growth rate, and species-specific response to environmental stochasticity (fourth row). The columns (a)-(c) have different (true) strengths of density regulation: $\gamma = 0.05$, 0.1 and 0.5, respectively, while the x-axis is the true proportion of variance due to within-species variation. The box-plots are point estimates from 1000 simulations, and the red asterisks indicate the true values. Results for common environmental effect $\sigma_c^2 = 0.01$ shown.

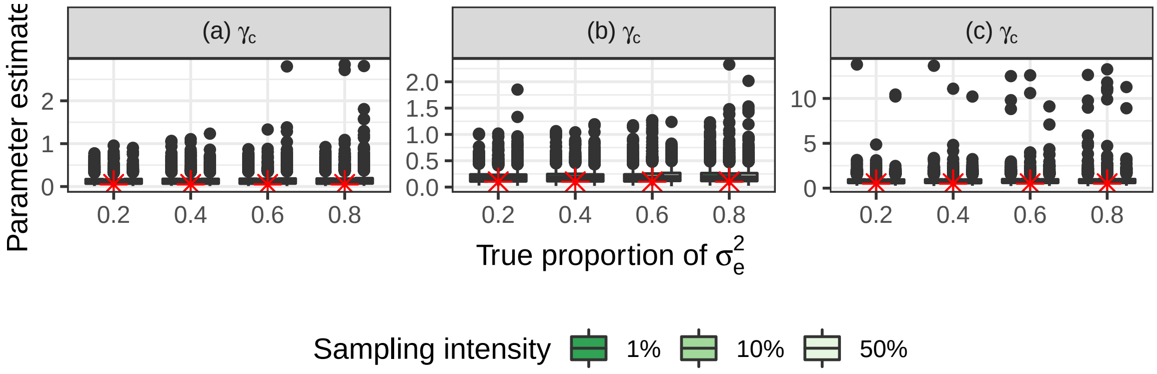

Figure S5: Estimates of strength of density regulation in general environment for common environmental effect equal to 0.1 from fitting a GLMM to simulated discrete population data. The x-axis is the true proportion of variance due to within-species variation. The box-plots are point estimates from 1000 simulations, and the red asterisks indicate the true values. Each column represents different strengths of density regulation, where γ and γ_c are both equal to 0.05, 0.1 and 0.5 from (a) to (c), respectively.

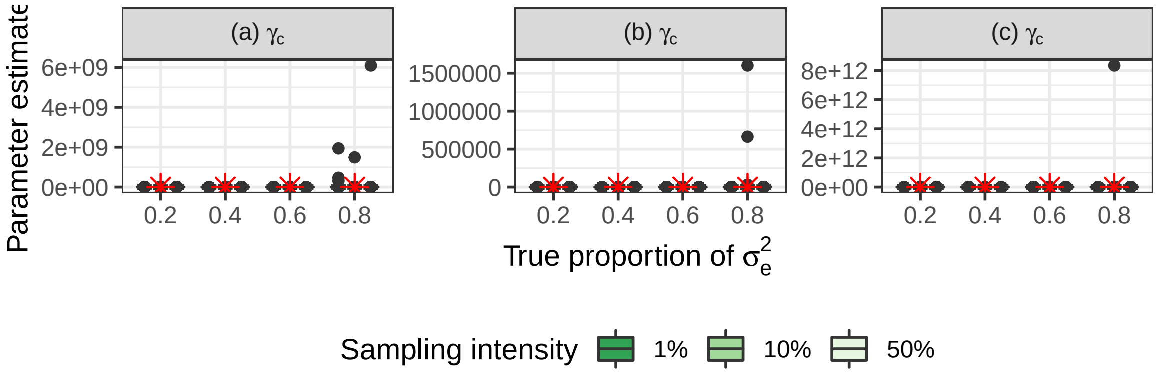

Figure S6: Estimates of strength of density regulation in general environment for common environmental effect equal to 0.01 from fitting a GLMM to simulated discrete population data. The x-axis is the true proportion of variance due to within-species variation. The box-plots are point estimates from 1000 simulations, and the red asterisks indicate the true values. Each column represents different strengths of density regulation, where γ and γ_c are both equal to 0.05, 0.1 and 0.5 from (a) to (c), respectively.

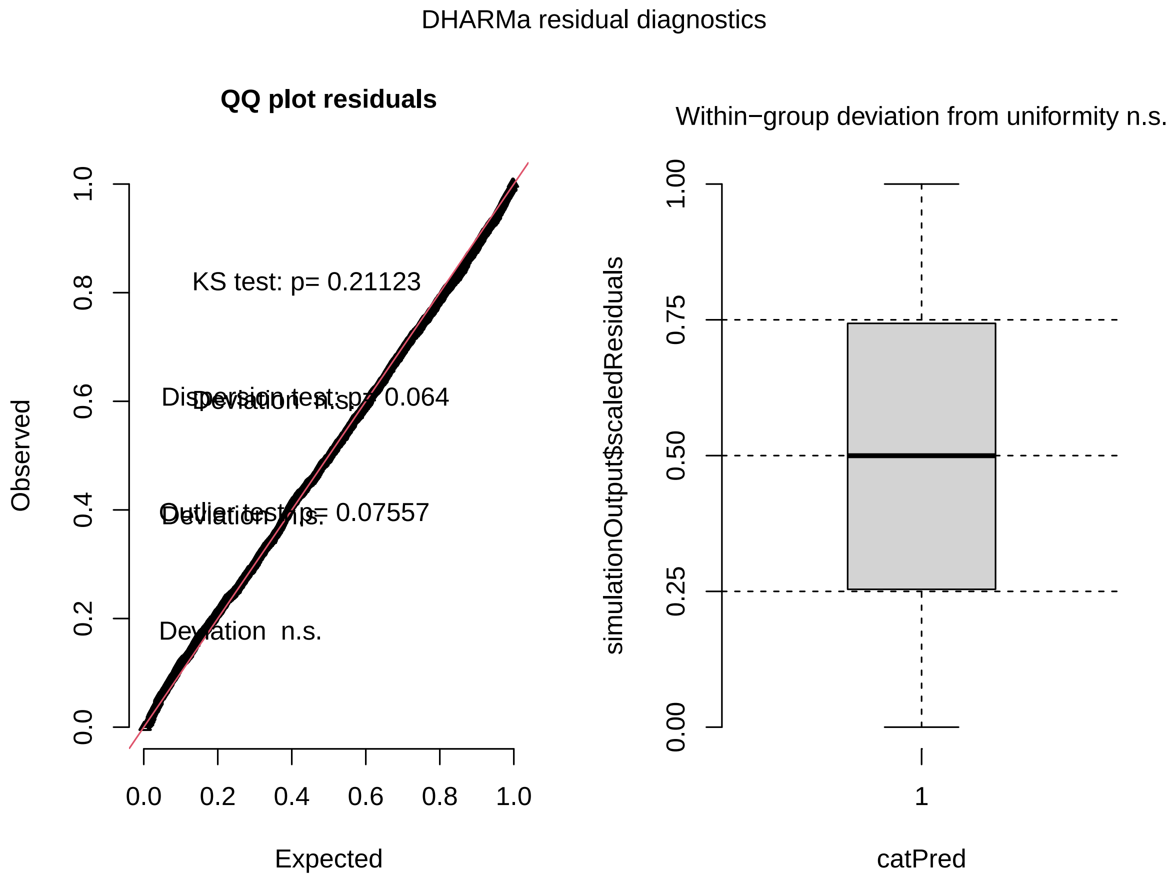

Figure S7: Residual plot for temporal model of fish data using the DHARMa package.

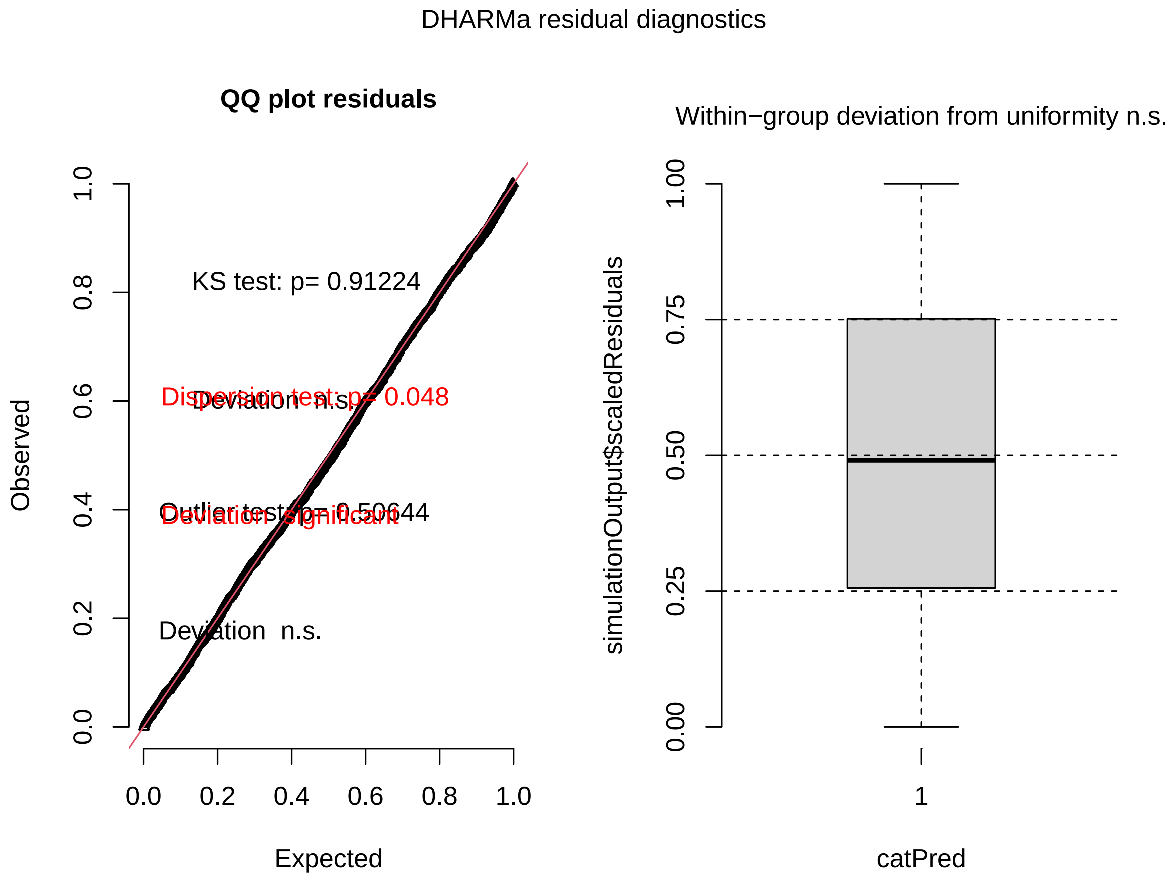

Figure S8: Residual plot for spatial model of fish data using the DHARMa package.
