# Supplementary material for: Analyzing dynamic species abundance distributions using generalized linear mixed models
Source: Ecology. 2022 Jun 23;103(9):e3742. doi: 10.1002/ecy.3742 (PMC9541646; doi:10.1002/ecy.3742)
Supplement: Supplementary file 6 — Appendix S6 [file ECY-103-e3742-s002.pdf]

Supporting Information for "Analyzing dynamic species abundance distributions using generalized linear mixed models" in Ecology by Erik Blystad Solbu, Bert van der Veen, Ivar Herfindal and Knut Anders Hovstad.

Appendix S6: Fish data case study

Contents

Introduction and data preparation **1**

Model fitting **3**
- Temporal model . . . 3
  - Variation in relative abundance . . . 3
  - Variation in mean log abundance . . . 4
    - A note on estimating variation in mean log abundance . . . 4
  - Specifiying data set . . . 4
  - Observation model . . . 4
- Spatial model . . . 5
  - Variation in relative abundance . . . 5
  - Variation in mean log abundance . . . 5
- Parameter uncertainty - bootstrap . . . 5

Results **7**
- Retrieving estimates . . . 7
- Summary table . . . 9
- Figures . . . 10
  - Correlation functions . . . 10
  - Total variance and partitioning . . . 14
  - Final figure . . . 16


```r
# Packages used
library(tidyverse)
library(patchwork)
library(sf)
library(glmmTMB)
# Optional packages for parallel computing of bootstrap estimates:
library(foreach)
library(parallel)
library(doParallel)
```

Introduction and data preparation

In this vignette, we analyze the "fish data" from the main text. This data set is downloaded from https://doi.org/10.5281/zenodo.5026943 where the "BioTIMEQuery_24_06_2021.zip" is the file that contains this and many other data sets. The `STUDY_ID` of the fish data is 467 and the following code loads the data set (which has been unzipped into `biotime` folder) and selects the data set and then writes this data set back to the working folder for easy access later.

```r
BioTIMEQuery_24_06_2021 <- read_csv("biotime/BioTIMEQuery_24_06_2021.csv")

fish_data <- BioTIMEQuery_24_06_2021 %>%
  filter(STUDY_ID == "467") %>%
  rename(ABUNDANCE = sum.allrawdata.ABUNDANCE,
         BIOMASS = sum.allrawdata.BIOMASS)
```

```r
write.csv(fish_data[, -1], "fish_data.csv", row.names = FALSE)
```

```r
# Here is also a direct link to the data set, but note that the code below uses
# the unzipped data file above:
# https://biotime.st-andrews.ac.uk/selectStudy.php?study=467

# Load the saved (much smaller) data set
fish_data <- read_csv("fish_data.csv")
```

When analyzing spatial correlation, we compute the distance between locations. To avoid numerical issues, we change the distance from meters to kilometers.

```r
# Transform to utm coordinates for spatial distance calculation
latlong_fish <- distinct(dplyr::select(fish_data, LATITUDE, LONGITUDE))

st_fish <- st_sfc(st_multipoint(cbind(latlong_fish$LONGITUDE,
                                      latlong_fish$LATITUDE)))

st_crs(st_fish) <- "+proj=longlat +datum=WGS84"

st_fish <- st_transform(st_fish, "+proj=utm +zone=29 ellps=WGS84")

utm_fish <- st_coordinates(st_fish)

# Convert distance to kilometers
ll_utm_fish <- as_tibble(cbind(latlong_fish, utm_fish[, -3]/1000))

fish_data <- left_join(fish_data, ll_utm_fish)

# Create spatial and temporal variables used by glmmTMB
fish_data <- fish_data %>%
  mutate(location = numFactor(X, Y),
         time_y = numFactor(YEAR))

# Cleanup
rm(list = c("latlong_fish", "ll_utm_fish", "st_fish", "utm_fish"))
```

This data set contains non-zero abundance observations of species. We choose to "fill in" the zeros which are implicit in the data set with the code below. Alternatively, a non-zero observational distribution can be used, such as the truncated Poisson distribution.

```r
# Wide transformation of the data and fill in zero abundance for each
# unobserved combination of location and time for each species.
fish_data_wide <- fish_data %>%
  dplyr::select(STUDY_ID, YEAR, time_y, location, GENUS_SPECIES, ABUNDANCE) %>%
  pivot_wider(names_from = GENUS_SPECIES,
              values_from = ABUNDANCE,
              values_fill = 0)

# Transform back to long format for analysis.
fish_data_long <- fish_data_wide %>%
  pivot_longer(cols = -c(STUDY_ID:location),
               names_to = "species",
               values_to = "abundance") %>%
  rename(year = YEAR)
```

```r
fish_data <- fish_data_long

# Cleanup
rm(list = c("fish_data_wide", "fish_data_long"))
```

Model fitting

This data set contains observations of abundance at different locations and time points. We are interested in modelling the variation and correlation in abundance of species. In this example we assume that the dynamics have a spatial component within a given time point and a temporal component within a given location. This means that we group observations by location when studying temporal dynamics, and group observations by time points when studying spatial dynamics. Splitting up the analysis in two parts, one temporal model and one spatial model is based on a simplifying assumption, and might not be advisable in all cases, for instance over very large spatial scales. An alternative is to consider models that allows for joint space-time correlation.

Temporal model

The following code analyze the temporal dynamics of the fish community:

```r
# Temporal model
fish_mod01 <- glmmTMB(abundance ~
                        # Relative log abundance dynamics:
                        # Within-species variation:
                        ou(time_y + 0 | species:location) +
                        # Among-species variation:
                        (1 | species) +
                        # Mean log abundance dynamics:
                        # Within-location variation:
                        ou(time_y + 0| location),
                      # Data set:
                      data = fish_data,
                      # Observation process:
                      family = poisson(link = "log"))
```

The model consists of the following elements:

```r
fish_mod01 <- glmmTMB(abundance ~
```

This is the dependent variable, $y_i(t, z)$, the observed measure of abundance, in this case counts.

Variation in relative abundance

```r
                        ou(time_y + 0 | species:location) +
```

This is the within-species variation, the first element describing variation in relative log abundance x_i. The specification of `species:location` means that at each location, we consider the variation of each species, and we consider this variation over time, i.e. `time_y + 0`. The variation over time has a covariance structure that follows an Ornstein-Uhlenbeck process, specified by `ou(...|...)`. Mathematically, this term is $e_i(t) \sim N(0, \sigma_e^2)$, or in vector form

$$\boldsymbol{b}_e \sim N(\boldsymbol{0}, \sigma_e^2 \boldsymbol{I}_L \otimes \boldsymbol{I}_S \otimes \boldsymbol{\rho}), \qquad \text{(Eq. S13)}$$

where $\boldsymbol{I}_L$ and $\boldsymbol{I}_S$ are identity matrices of order L and S, where L and S are the number of locations and species in the data set, respectively. $\boldsymbol{\rho}$ is a $T \times T$ diagonal matrix, where T is the number of time points, with elements $\rho_{kl} = e^{-\gamma|t_l - t_k|}$ and γ is a parameter we estimate. Note that the time points do not have to be

equidistant. In the fish data set we have $t = 1987, 1996, 1998, 1999$, meaning that the different values of $|t_l - t_k|$ are 0, 1, 2, 3, 9, 11, 12, and $\boldsymbol{\rho}$ becomes

$$\boldsymbol{\rho} = \begin{bmatrix} e^{-\gamma\cdot 0} & & & \\ e^{-\gamma\cdot 9} & e^{-\gamma\cdot 0} & & \\ e^{-\gamma\cdot 11} & e^{-\gamma\cdot 2} & e^{-\gamma\cdot 0} & \\ e^{-\gamma\cdot 12} & e^{-\gamma\cdot 3} & e^{-\gamma\cdot 1} & e^{-\gamma\cdot 0} \end{bmatrix}. \qquad \text{(Eq. S14)}$$

Finally, $\otimes$ is the Kronecker product, meaning that we multiply each element of $\boldsymbol{I}_L$ with $\boldsymbol{I}_S$, and each element of that with $\boldsymbol{\rho}$:

$$\boldsymbol{I}_L \otimes \boldsymbol{I}_S \otimes \boldsymbol{\rho} = \begin{bmatrix} 1\cdot\boldsymbol{\rho} & 0\cdot\boldsymbol{\rho} & \dots & 0\cdot\boldsymbol{\rho} \\ 0\cdot\boldsymbol{\rho} & 1\cdot\boldsymbol{\rho} & \dots & 0\cdot\boldsymbol{\rho} \\ \vdots & & \ddots & \\ 0\cdot\boldsymbol{\rho} & 0\cdot\boldsymbol{\rho} & \dots & 1\cdot\boldsymbol{\rho} \end{bmatrix}, \qquad \text{(Eq. S15)}$$

meaning that $\boldsymbol{I}_L \otimes \boldsymbol{I}_S \otimes \boldsymbol{\rho}$ is a block diagonal matrix of size $LST \times LST$.

```
                    (1 | species) +
```

This is among-species variation, and the second element describing variation in relative log abundance x_i. The specification means that we assume the mean value of each species i across all locations and time points follows a distribution $h_i \sim N(0, \sigma_h^2)$, or in vector form $\boldsymbol{b}_h \sim N(\mathbf{0}, \sigma_h^2 \boldsymbol{I}_S)$, where $\boldsymbol{I}_S$ is the same identity matrix as above.

Variation in mean log abundance

```
                    ou(time_y + 0| location) +
```

This is the within-location variation used to describe temporal variation in mean log abundance $\bar{X}$. Following the description of within-species variation above, we assume that at each `location`, the mean abundance varies over time, `time_y + 0`, according to an Ornstein-Uhlenbeck process, `ou(...|...)`. Mathematically, we can denote this variation as $c(t) \sim N(0, \sigma_c^2)$, where subscript c denotes "common" environmental variation, and in vector form $\boldsymbol{b}_c \sim N(\mathbf{0}, \sigma_c^2 \boldsymbol{\rho}_c)$. The correlation matrix $\boldsymbol{\rho}_c$ is identical to the one defined above [equation (2)], but we estimate a different correlation parameter, γ_c, hence the subscript.

A note on estimating variation in mean log abundance Above, we have used a simple structure, in terms of number of parameters, to describe the variation in mean log abundance, but it can still be difficult to estimate due to few levels of variation, i.e. the number of locations. In some cases, where we are most interested in studying the dynamics of relative abundance, it might be more straightforward to account for variation in mean log abundance by using fixed effects. In the model described here, such a term could be:

```
                    time_y * location +
```

which would estimate the mean value of each combination of time and location. The most important thing is to account for differences in mean log abundance in some way or else the estimates of variation in relative abundance will be unreliable. Conversely, a more complicated structure, e.g. an unstructured covariance matrix, could also have been used do describe the variation in mean log abundance, but this would in turn require more data (locations and time points).

Specifiying data set

```
                  data = fish_data,
```

Observation model

```
                    family = poisson(link = "log"))
```

We assume the observation process follows a Poisson distribution. This is a modelling choice based on the assumption that there are implicit zeros in the data set, otherwise a model with a non-zero, integer-valued, observation process should be used, such as a truncated Poisson distribution.

Spatial model

The spatial model is describing the variation and correlation between communities across space within a single time point. The procedure is quite similar to the section on temporal analysis. The following code analyze the spatial dynamics of the fish community:

```
# Spatial model
fish_mod02 <- glmmTMB(abundance ~
                        # Relative log abundance dynamics:
                        # Within-species variation:
                        exp(location + 0 | species:time_y) +
                        # Among-species variation:
                        (1 | species) +
                        # Mean log abundance dynamics:
                        # Within-year variation:
                        exp(location + 0 | time_y),
                      # Data set:
                      data = fish_data,
                      # Observation process:
                      family = poisson(link = "log"))
```

We now highlight the changes from the temporal model.

Variation in relative abundance

```
                        exp(location + 0 | species:time_y) +
```

This is the (spatial) within-species variation. The specification `species:time_y` means that we consider variation in relative abundance of each species at a single time point, and the variation is across space `location + 0`. The structure of the correlation matrix is an exponential decay function. Mathematically, we have $e_i(z) \sim N(0, \sigma_e^2)$, or in vector form $\boldsymbol{b}_e \sim N(\boldsymbol{0}, \sigma_e^2 \boldsymbol{I}_T \otimes \boldsymbol{I}_S \otimes \boldsymbol{\rho})$, where $\boldsymbol{I}_T$ is the identity matrix of order T, and now $\boldsymbol{\rho}$ is a $L \times L$ diagonal matrix, with elements $\rho_{kl} = e^{-\alpha d(l,k)}$, where $d(l,k)$ is the distance between location l and k and α is the inverse of spatial scaling, $1/\alpha$.

Variation in mean log abundance

```
                        exp(location + 0 | time_y) +
```

This is within-time variation used to describe spatial variation in mean log abundance $\bar{X}$. The term is $c(z) \sim N(0, \sigma_c^2)$, and in vector form $\boldsymbol{b}_c \sim N(\boldsymbol{0}, \sigma_c^2 \boldsymbol{\rho}_c)$, where the correlation matrix $\boldsymbol{\rho}_c$ is similar to $\boldsymbol{\rho}$, but with a different spatial correlation parameters α_c, hence the subscript.

Parameter uncertainty - bootstrap

To calculate the parameter uncertainty, we use parametric bootstrap, i.e. simulating from the fitted model and re-estimate the parameters repeatedly, to generate sampling distributions. This also makes it easier to compute uncertainty in different functions of the estimated parameters that we are going to use later. The number of bootstrap replicates will determine the time it takes to run the following code.

```r
# Bootstrap

# Temporal model
boot_fish_est_01 <- c()
for (i in 1:100){
  # Simulate new data
  boot_data_fish <- fish_data %>%
    mutate(sim = simulate(fish_mod01)$sim_1)
  # Re-fit the model
  boot_fish_mod01 <- glmmTMB(sim ~
                              ou(time_y + 0 | species:location) +
                              (1 | species) +
                              ou(time_y + 0 | location),
                            data = boot_data_fish,
                            family = poisson(link = "log"))
  # Store the bootstrap estimates:
  boot_fish_est_01 <- rbind(boot_fish_est_01,
                           c(boot_fish_mod01$fit$par, i))
}
```

In order to increase the accuracy of the bootstrap without taking too long time, we do the bootstrap in parallel. Each model, temporal and spatial, (at 1000 iterations each) took roughly 30 minutes on a Intel i5-8350U with 8 logical processors.

```r
# Temporal model
f_sim_and_est_1 <- function(i){
  # Simulate new data
  boot_data_fish <- fish_data %>%
    mutate(sim = simulate(fish_mod01)$sim_1)
  # Re-fit the model
  boot_fish_mod01 <- glmmTMB(sim ~
                              ou(time_y + 0 | species:location) +
                              (1 | species) +
                              ou(time_y + 0 | location),
                            data = boot_data_fish,
                            family = poisson(link = "log"))
  # Return the estimated parameters
  c(boot_fish_mod01$fit$par, i)
}

# How many cores do we have available?
k <- detectCores()

# Use all cores except one
cl <- makeCluster(k - 1)
registerDoParallel(cl)

# Run the bootstrap in parallel (un-comment to run)
# boot_fish_est_01 <- foreach(i = 1:1000,
#                     .combine = "rbind",
#                     .errorhandling = "remove",
#                     .packages = c("tidyverse", "glmmTMB")) %dopar% {f_sim_and_est_1(i)}

# Shut down the workers
```

```
stopCluster(cl)
```

Similarly for the spatial model, we simulate and refit the model:

```
# Spatial model
f_sim_and_est_2 <- function(i){
  # Simulate new data
  boot_data_fish <- fish_data %>%
    mutate(sim = simulate(fish_mod02)$sim_1)
  # Re-fit the model
  boot_fish_mod02 <- glmmTMB(sim ~
                               exp(location + 0 | species:time_y) +
                               (1 | species) +
                               exp(location + 0 | time_y),
                             data = boot_data_fish,
                             family = poisson(link = "log"))
  # Return the estimated parameters
  c(boot_fish_mod02$fit$par, i)
}

# How many cores do we have available?
k <- detectCores()

# Use all cores except one
cl <- makeCluster(k - 1)
registerDoParallel(cl)

# Run the bootstrap in parallel (un-comment to run)
# boot_fish_est_02 <- foreach(i = 1:1000,
#                      .combine = "rbind",
#                      .errorhandling = "remove",
#                      .packages = c("tidyverse", "glmmTMB")) %dopar% {f_sim_and_est_2(i)}

# Shut down the workers
stopCluster(cl)
```

```
# Save bootstrap for easy access if needed (un-comment the first time you run the
# bootstrap)
# write_rds(list(boot_fish_est_01,
#                boot_fish_est_02), "fish_boot_vignette.rds")
# Load bootstrap if needed
boot <- read_rds("fish_boot_vignette.rds")
boot_fish_est_01 <- boot[[1]]
boot_fish_est_02 <- boot[[2]]
```

Results

Retrieving estimates

The point estimates can be returned from the model using `'model'$fit$par`. The number of parameters depends on the model specification and below we summarize the parameters included by each term.

Model dynamics	R term	Parameters
Relative log abundance	`ou(time + 0 | species:location)`	$\ln(\sigma_e)$
		$\ln(1/\gamma)$
	`exp(location + 0 | species:time)`	$\ln(\sigma_e)$
		$\ln(1/\alpha)$
	`(1 | species)`	$\ln(\sigma_h)$
Mean log abundance	`ou(time + 0 | location)`	$\ln(\sigma_c)$
		$\ln(1/\gamma_c)$
	`exp(location + 0 | time)`	$\ln(\sigma_c)$
		$\ln(1/\alpha_c)$

For instance, if the model specification is

```
ou(time_y + 0 | species:location) +
(1 | species) +
ou(time_y + 0| location)
```

the parameters returned would be $[\beta_0, \ln(\sigma_e), \ln(1/\gamma), \ln(\sigma_h), \ln(\sigma_c), \ln(1/\gamma_c)]$, based on the order of the terms and the intercept β_0 is included (first) without specification. We are also interested in the following dynamic parameters, which are functions of the model parameters:

Definition	Description
$\sigma_s^2 = \sigma_e^2 \cdot 2\gamma$	Species specific response to environmental variation
$\sigma_r^2 = \sigma_h^2 \cdot \gamma^2$	Variation in growth rate among species
$\sigma_E^2 = \sigma_c^2 \cdot 2\gamma_c$	General response to (temporal) environmental variation
$1/\gamma$	Temporal scale
$1/\alpha$	Spatial scale

With the following code we transform the model output to parameters of interest.

```
# Join point estimates with bootstrap replicates:
fish_est_01 <- rbind(c(fish_mod01$fit$par, 0),
                     boot_fish_est_01)
fish_est_02 <- rbind(c(fish_mod02$fit$par, 0),
                     boot_fish_est_02)
# Set column names. Note 'rep = 0' are the point estimates.
colnames(fish_est_01) <- c("beta0", "lse2", "lgamma", "lsh2",
                           "lsc2", "lgamma_c", "rep")
colnames(fish_est_02) <- c("beta0", "lse2", "lalphainv", "lsh2",
                           "lsc2", "lalpha_cinv", "rep")

fish_est_01 <- as_tibble(fish_est_01)
fish_est_02 <- as_tibble(fish_est_02)

# Transform parameters
fish_est_01 <- fish_est_01 %>%
  mutate(se2 = exp(lse2) ^ 2,
         gamma = exp(lgamma),
         gamma_c = exp(lgamma_c),
         sh2 = exp(lsh2) ^ 2,
         sc2 = exp(lsc2) ^ 2,
         ss2 = se2 * 2 * gamma,
         sr2 = sh2 * gamma ^ 2)

fish_est_02 <- fish_est_02 %>%
  mutate(se2 = exp(lse2) ^ 2,
         alpha = 1 / exp(lalphainv),
```

```
         alpha_c = 1 / exp(lalpha_cinv),
         sh2 = exp(lsh2) ^ 2,
         sc2 = exp(lsc2) ^ 2)
```

Summary table

With the transformation done, we can make a table that summarize the point estimates and bootstrap confidence intervals.

```
# Select point estimates (rep == 0) for temporal and spatial model
fish_est <- bind_rows(add_column(filter(fish_est_01, rep == 0),
                                 dim = "Temporal"),
                      add_column(filter(fish_est_02, rep == 0),
                                 dim = "Spatial")) %>%
  # Compute temporal (tr) and spatial (sp) scale, for relative log abundance
  # and mean log abundance (_c)
  mutate(tr = 1 / gamma,
         sp = 1 / alpha,
         tr_c = 1 / gamma_c,
         sp_c = 1 / alpha_c) %>%
  dplyr::select(dim, sh2, se2, tr, sp,
                sc2, tr_c, sp_c,
                gamma, ss2, sr2) %>%
  pivot_longer(sh2:sr2) %>%
  filter(!is.na(value)) %>%
  # Re-arrange the parameters
  add_column(order = c(1, 3, 5, 7, 9, 11:13, 2, 4, 6, 8, 10)) %>%
  arrange(order)

# Make confidence intervals for bootstrap estimates (rep != 0)
fish_conf_int <- bind_rows(add_column(filter(fish_est_01, rep != 0),
                                      dim = "Temporal"),
                           add_column(filter(fish_est_02, rep != 0),
                                      dim = "Spatial")) %>%
  # Compute temporal (tr) and spatial (sp) scale, for relative log abundance
  # and mean log abundance (_c)
  mutate(tr = 1 / gamma,
         sp = 1 / alpha,
         tr_c = 1 / gamma_c,
         sp_c = 1 / alpha_c) %>%
  dplyr::select(dim, sh2, se2, tr, sp,
                sc2, tr_c, sp_c,
                gamma, ss2, sr2) %>%
  pivot_longer(sh2:sr2) %>%
  filter(!is.na(value)) %>%
  group_by(dim, name) %>%
  summarise(conf1 = quantile(value, 0.025),
            conf2 = quantile(value, 0.975))

# Make confidence intervals a single variable
fish_res <- left_join(fish_est, fish_conf_int, by = c("dim", "name")) %>%
  mutate(`95% CI` = paste("(", round(conf1, digits = 2), ", ",
                          round(conf2, digits = 2), ")", sep = ""))
```

```r
# Add names and organize parameters
fish_res_names <- fish_res %>%
  dplyr::select(dim, name) %>%
  add_column(` ` = c("Within samples", "", "", "", "", "",
                     "Among samples", "", "", "",
                     "Dynamic parameters", "", ""),
             Parameter = c("Among species - ecological heterogeneity, $\\sigma_h^2$",
                           "",
                           "Within species - environmental effects, $\\sigma_e^2$",
                           "",
                           "Temporal scale, $1/\\gamma$",
                           "Spatial scale, $1/\\alpha$",
                           "General environment, $\\sigma_c^2$",
                           "",
                           "Temporal scale, $1/\\gamma_c$",
                           "Spatial scale, $1/\\alpha_c$",
                           "Strength of density regulation, $\\gamma$",
                           "Species-specific response to environemnt, $\\sigma_s^2$",
                           "Variation in growth rate among species, $\\sigma_r^2$"))

fish_res <- left_join(fish_res, fish_res_names, by = c("dim", "name"))

# Keep relevant variables
fish_res_final <- fish_res %>%
  mutate(Estimate = round(value, digits = 2)) %>%
  dplyr::select(` `, Parameter, Estimate, `95% CI`)
```

```r
# Render table
knitr::kable(fish_res_final)
```

	Parameter	Estimate	95% CI
Within samples	Among species - ecological heterogeneity, σ_h^2	9.00	(5.37, 11.93)
		8.51	(4.65, 11.06)
	Within species - environmental effects, σ_e^2	5.08	(4.25, 5.64)
		5.14	(4.35, 5.75)
	Temporal scale, $1/\gamma$	5.78	(4.67, 7.2)
	Spatial scale, $1/\alpha$	8.57	(7.12, 10.68)
Among samples	General environment, σ_c^2	1.11	(0.36, 1.64)
		0.99	(0.45, 1.42)
	Temporal scale, $1/\gamma_c$	13.67	(3.13, 31.55)
	Spatial scale, $1/\alpha_c$	2.76	(0.18, 6.15)
Dynamic parameters	Strength of density regulation, γ	0.17	(0.14, 0.21)
	Species-specific response to environemnt, σ_s^2	1.76	(1.41, 2.01)
	Variation in growth rate among species, σ_r^2	0.27	(0.14, 0.42)

Figures

Correlation functions

We want to plot the temporal and spatial correlation in relative log abundance, and in mean log abundance. Therefore, we need to compute the following functions for a range of time points, for each bootstrap replicate

and original point estimates:

$$\text{Temporal correlation in relative log abundance:} \quad \rho_x(t) = \frac{\sigma_e^2 e^{-\gamma t} + \sigma_h^2}{\sigma_e^2 + \sigma_h^2} \qquad \text{(Eq. S16)}$$

$$\text{Spatial correlation in relative log abundance:} \quad \rho_x(d) = \frac{\sigma_e^2 e^{-\alpha d} + \sigma_h^2}{\sigma_e^2 + \sigma_h^2}, \qquad \text{(Eq. S17)}$$

$$\text{Temporal correlation in mean log abundance:} \quad \rho_{\bar{X}}(t) = e^{-\gamma_c t}, \qquad \text{(Eq. S18)}$$

$$\text{Spatial correlation in mean log abundance:} \quad \rho_{\bar{X}}(d) = e^{-\alpha_c d}, \qquad \text{(Eq. S19)}$$

where d is distance. In the following we compute these four correlations:

```
# Temporal correlation in relative log abundance
# Time points to compute correlation for
time = seq(0, max(fish_data$year) - min(fish_data$year), by = 1)
# Correlation function
f_temp_x_cor <- function(data){
  tibble(time = time,
         cor = (data$se2 * exp(-data$gamma * time) + data$sh2) /
           (data$se2 + data$sh2))
}
# Compute correlation for each replicate
fish_cor_01_x <- fish_est_01 %>%
  group_by(rep) %>%
  nest() %>%
  mutate(cor = map(data, f_temp_x_cor)) %>%
  dplyr::select(-data) %>%
  unnest(cor) %>%
  ungroup()
```

```
# Temporal correlation in mean log abundance
# Time points to compute correlation for
time <- seq(0, max(fish_data$year) - min(fish_data$year), by = 1)
# Correlation function
f_temp_xbar_cor <- function(data){
  tibble(time = time,
         cor = exp(-data$gamma_c))
}
# Compute correlation for each replicate
fish_cor_01_xbar <- fish_est_01 %>%
  group_by(rep) %>%
  nest() %>%
  mutate(cor = map(data, f_temp_xbar_cor)) %>%
  dplyr::select(-data) %>%
  unnest(cor) %>%
  ungroup()
```

```
# Spatial correlation in relative log abundance
# Distances to compute correlation for
distance = seq(0, round(max(dist(parseNumLevels(fish_data$location)))), by = 1)
# Correlation function
f_space_x_cor <- function(data){
  distance = distance
  tibble(distance = distance,
         cor = (data$se2 * exp(- data$alpha * distance) + data$sh2) /
```

```
           (data$se2 + data$sh2))
}
# Compute correlation for each replicate
fish_cor_02_x <- fish_est_02 %>%
  group_by(rep) %>%
  nest() %>%
  mutate(cor = map(data, f_space_x_cor)) %>%
  dplyr::select(-data) %>%
  unnest(cor) %>%
  ungroup()
```

```
# Spatial correlation in mean log abundance
# Distances to compute correlation for
distance = seq(0, round(max(dist(parseNumLevels(fish_data$location)))), by = 1)
# Correlation function
f_space_xbar_cor <- function(data){
  distance = distance
  tibble(distance = distance,
         cor = exp(- data$alpha_c * distance))
}
# Compute the correlation for each replicate
fish_cor_02_xbar <- fish_est_02 %>%
  group_by(rep) %>%
  nest() %>%
  mutate(cor = map(data, f_space_xbar_cor)) %>%
  dplyr::select(-data) %>%
  unnest(cor) %>%
  ungroup()
```

Then we compute the 95% confidence intervals of each correlation function for the bootstrap estimates:

```
# Temporal correlation in relative log abundance
fish_cor_01_x_sum <- fish_cor_01_x %>%
  # Skip the initial point estimates
  filter(rep != 0) %>%
  group_by(time) %>%
  summarise(q025 = quantile(cor, probs = 0.025),
            q975 = quantile(cor, probs = 0.975))

# Temporal correlation in mean log abundance
fish_cor_01_xbar_sum <- fish_cor_01_xbar %>%
  # Skip the initial point estimates
  filter(rep != 0) %>%
  group_by(time) %>%
  summarise(q025 = quantile(cor, probs = 0.025),
            q975 = quantile(cor, probs = 0.975))

# Spatial correlation in relative log abundance
fish_cor_02_x_sum <- fish_cor_02_x %>%
  # Skip the initial point estimates
  filter(rep != 0) %>%
  group_by(distance) %>%
  summarise(q025 = quantile(cor, probs = 0.025),
            q975 = quantile(cor, probs = 0.975))
```

```r
# Spatial correlation in mean log abundance
fish_cor_02_xbar_sum <- fish_cor_02_xbar %>%
  # Skip the initial point estimates
  filter(rep != 0) %>%
  group_by(distance) %>%
  summarise(q025 = quantile(cor, probs = 0.025),
            q975 = quantile(cor, probs = 0.975))
```

We can make figures of each correlation function, where the line are the estimates from the data set and the grey ribbon illustrates the confidence interval.

```r
# Temporal correlation in relative log abundance
p1 <- fish_cor_01_x_sum %>%
  ggplot(aes(x = time, y = NULL)) +
  geom_ribbon(aes(x = time, ymin = q025, ymax = q975), alpha = 0.25) +
  geom_line(data = filter(fish_cor_01_x, rep == 0),
            aes(x = time, y = cor)) +
  lims(y = c(0, 1)) +
  labs(x = "Time (years)", y = "Correlation") +
  scale_x_continuous(breaks = seq(0, 12, by = 3)) +
  theme_bw()

# p1 # un-comment to view the figure separately
```

```r
# Spatial correlation in relative log abundance
p2 <- fish_cor_02_x_sum %>%
  ggplot(aes(x = distance, y = NULL)) +
  geom_ribbon(aes(x = distance, ymin = q025, ymax = q975), alpha = 0.25) +
  geom_line(data = filter(fish_cor_02_x, rep == 0),
            aes(x = distance, y = cor)) +
  lims(y = c(0, 1)) +
  labs(x = "Distance (km)", y = "Correlation") +
  scale_x_continuous(breaks = seq(0, 36, by = 6)) +
  theme_bw()

# p2 # un-comment to view the figure separately
```

```r
# Temporal correlation in mean log abundance
p3 <- fish_cor_01_xbar_sum %>%
  ggplot(aes(x = time, y = NULL)) +
  geom_ribbon(aes(x = time, ymin = q025, ymax = q975), alpha = 0.25) +
  geom_line(data = filter(fish_cor_01_xbar, rep == 0),
            aes(x = time, y = cor)) +
  lims(y = c(0, 1)) +
  labs(x = "Time (years)", y = "Correlation") +
  scale_x_continuous(breaks = seq(0, 12, by = 3)) +
  theme_bw()

# p3 # un-comment to view the figure separately
```

```r
# Spatial correlation in mean log abundance
p4 <- fish_cor_02_xbar_sum %>%
  ggplot(aes(x = distance, y = NULL)) +
  geom_ribbon(aes(x = distance, ymin = q025, ymax = q975), alpha = 0.25) +
  geom_line(data = filter(fish_cor_02_xbar, rep == 0),
```

```
           aes(x = distance, y = cor)) +
  lims(y = c(0, 1)) +
  labs(x = "Distance (km)", y = "Correlation") +
  scale_x_continuous(breaks = seq(0, 36, by = 6)) +
  theme_bw()

# p4 # un-comment to view the figure separately
```

Total variance and partitioning

We are also interested in the estimated total variance in relative log abundance, $\sigma_x^2 = \sigma_e^2 + \sigma_h^2$ and mean log abundance $\sigma_{\bar{X}}^2 = \sigma_c^2$, and the uncertainty. In addition, we can compute the relative contribution of environmental effects and heterogeneity among species on the total variance in relative log abundance. We do this for both the temporal model and spatial model.

```
# Total variance in the temporal model
tot_var_sum_01 <- fish_est_01 %>%
  mutate(boot = as.numeric(rep > 0),
         rel_tot_var = se2 + sh2,
         gen_tot_var = sc2,
         prc_rel_se2 = se2 / rel_tot_var,
         prc_rel_sh2 = sh2 / rel_tot_var) %>%
  dplyr::select(boot:prc_rel_sh2) %>%
  pivot_longer(rel_tot_var:prc_rel_sh2) %>%
  group_by(boot, name) %>%
  summarise(est = mean(value),
            q025 = quantile(value, probs = 0.025),
            q975 = quantile(value, probs = 0.975)) %>%
  add_column(dimension = "Temporal") %>%
  mutate(environment = ifelse(str_detect(name, "rel"), "Species specific", "General"),
         tot_var = ifelse(str_detect(name, "tot"), "Total variance", "Relative"),
         het = ifelse(str_detect(name, "_sh"), "Heterogeneity", "Environmental"))

# Total variance in the spatial model
tot_var_sum_02 <- fish_est_02 %>%
  mutate(boot = as.numeric(rep > 0),
         rel_tot_var = se2 + sh2,
         gen_tot_var = sc2,
         prc_rel_se2 = se2 / rel_tot_var,
         prc_rel_sh2 = sh2 / rel_tot_var) %>%
  dplyr::select(boot:prc_rel_sh2) %>%
  pivot_longer(rel_tot_var:prc_rel_sh2) %>%
  group_by(boot, name) %>%
  summarise(est = mean(value),
            q025 = quantile(value, probs = 0.025),
            q975 = quantile(value, probs = 0.975)) %>%
  add_column(dimension = "Spatial") %>%
  mutate(environment = ifelse(str_detect(name, "rel"), "Species specific", "General"),
         tot_var = ifelse(str_detect(name, "tot"), "Total variance", "Relative"),
         het = ifelse(str_detect(name, "_sh"), "Heterogeneity", "Environmental"))

tot_var_sum <- bind_rows(tot_var_sum_01,
                         tot_var_sum_02)
```

```
dodge <- position_dodge(width = 0.9)

tot_var <- tot_var_sum %>%
  filter(tot_var == "Total variance")

p5 <- tot_var %>%
  ggplot(aes(x = dimension, y = est)) +
  geom_col(data = filter(tot_var,
                         boot == 0),
           aes(linetype = dimension),
           colour = "black", fill = "dark grey") +
  geom_errorbar(data = filter(tot_var,
                              boot == 1),
                aes(ymin = q025, ymax = q975, linetype = dimension),
                position = dodge, width = 0.25) +
  scale_linetype_manual(values = c("Spatial" = 1,
                                   "Temporal" = 1)) +
  labs(x = "Dimension", y = "Total variance") +
  guides(linetype = "none") +
  facet_grid(. ~ environment) +
  theme_bw()

# p5 # un-comment to view figure separately
```

```
dodge <- position_dodge(width = 1)

rel_var <- tot_var_sum %>%
  filter(tot_var == "Relative")

p6 <- rel_var %>%
  ggplot(aes(x = het, y = est)) +
  geom_col(data = filter(rel_var,
                         boot == 0),
           aes(fill = het, linetype = dimension),
           colour = "black", position = dodge) +
  geom_errorbar(data = filter(rel_var,
                              boot == 1),
                aes(ymin = q025, ymax = q975, linetype = dimension),
                position = dodge, width = 0.25) +
  scale_fill_manual(values = c("Heterogeneity" = "#8dd3c7",
                               "Environmental" = "#bebada")) +
  scale_x_discrete(labels = c("Env.", "Het.")) +
  scale_linetype_manual(values = c("Spatial" = 1,
                                   "Temporal" = 1)) +
  labs(x = "Variance component", y = "Proportion") +
  lims(y = c(0, 1)) +
  guides(fill = "none",
         linetype = "none") +
  facet_grid(. ~ environment) +
  theme_bw()

# p6 # un-comment to view figure separately
```

Final figure

The following code displays the different results in one figure.

```
(((p1 + p2) / (p3 | p4)) / (p5 | p6)) + plot_annotation(tag_levels = "a")
```

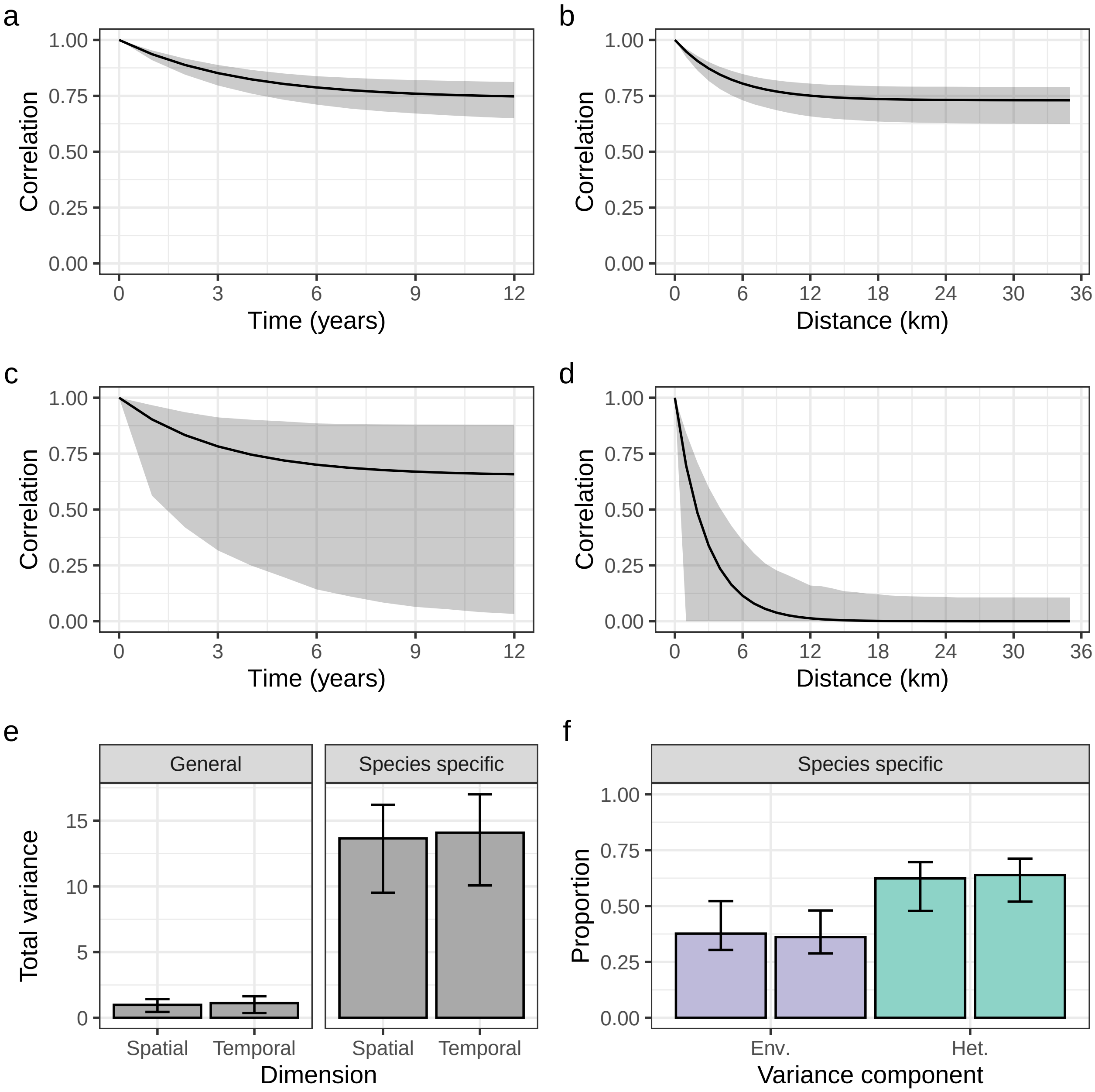

Figure S1: Analysis of fish community data from Ria de Aveiro, Portugal. Temporal (a) and spatial (b) correlation of relative log abundance with gray bands as 95% confidence intervals. Temporal (c) and spatial (d) correlation of mean log abundance with gray bands as 95% confidence intervals. (e) Estimated total variance for mean log abundance (general environmental effects) and relative log abundance (species-specific effects) with 95% confidence intervals. (f) Proportion of the total variance attributed to temporal environmental variation within species (Env.), and among species variation or ecological heterogeneity, (Het.) with 95% confidence intervals. For each variance component the columns are from the spatial (left) and temporal (right) model. All confidence intervals are based on 1000 bootstrap replicates.
